# DNA Damage and Repair in Ovarian Cancer: Focus on MicroRNAs

**DOI:** 10.3390/cancers17183011

**Published:** 2025-09-15

**Authors:** Katarzyna D. Arczewska, Agnieszka Piekiełko-Witkowska

**Affiliations:** Department of Biochemistry and Molecular Biology, Centre of Postgraduate Medical Education, 01-813 Warsaw, Poland

**Keywords:** microRNA, ovarian cancer, DNA repair, PARP1, olaparib, BRCA1/2

## Abstract

Ovarian cancer (OvCa) is one of the most life-threatening female malignancies that affects 300,000 women annually worldwide. Impaired mechanisms of DNA repair are the leading cause of mutations underlying the OvCa development. microRNAs are short non-coding RNAs that regulate the expression of genes by binding to their transcripts and inducing mRNA degradation or inhibition of translation. Here, we review the miRNA-mediated dysregulation of genes involved in DNA damage response (DDR) and DNA repair pathways in OvCa. Apparently, miRNAs are capable of targeting the crucial mediators of DDR (e.g., miR-203a-3p targeting ATM (Ataxia Telangiectasia Mutated)), homologous repair (such as BRCA1 targeted by miR-9, miR-1255b, miR-193b, and miR-148b), non-homologous end joining (with RNF8 being regulated by miR-214), nucleotide excision repair (involving DDB2 targeted by miR-328-3p), or translesion DNA synthesis (involving RAD18, participating also in homologous repair and targeted by miR-379-5p). We also discuss miRNAs (such as miR-519a-3p, let-7e, miR-216b), which affect responses to OvCa therapy by targeting PARP1 (Poly(ADP-Ribose) Polymerase-1). Finally, we also discuss why, despite the identification of multiple miRNAs capable of regulating DNA repair genes, as well as those involved in the response to therapy, no miRNA-based drugs have been approved for OvCa treatment in clinics.

## Simple Summary

DNA repair mechanisms are crucial for maintanence of DNA and cell integrity. When disrupted, they can lead to increased mutation rate and contribute to the cancerogenesis. On the other hand, cancer cells that bear mutations in genes involved in DNA repair are more prone to DNA damaging insults which opens opportunities for treatments involving synthetic lethality. microRNAs are short non-coding RNAs that regulate expression by binding to gene transcripts and inducing mRNA degradation or inhibition of translation. Here, we review the miRNA-mediated dysregulation of genes involved in DNA damage response (DDR) and DNA repair pathways in ovarian cancer (OvCa), one of the deadliest female malignancy. We also discuss miRNAs, which affect response to OvCa therapy by regulating PARP1 (Poly(ADP-Ribose) Polymerase-1), a central gene of the OvCa synthetic lethality treatments. Finally, we address the limitations of miRNAs as diagnostic biomarkers and potential targets for therapeutic interventions.

## 1. Introduction

Ovarian cancer (OvCa) is among the deadliest malignancies, affecting over 300,000 women annually worldwide and resulting in more than 200,000 deaths each year [1,2]. The prognosis for patients with OvCa remains poor, with a five-year survival rate below 40% [3]. The most common and aggressive histological subtype is high-grade serous ovarian carcinoma (HGSOC), originating primarily from the epithelium of the fallopian tubes [4], which accounts for approximately 70–80% of OvCa-related deaths. Less frequent subtypes include endometrioid OvCa (~10%) and clear cell OvCa (~6%), both of which are thought to arise from the endometrium. The rarest histotypes include low-grade serous and mucinous ovarian cancers, each contributing approximately 5% of cases. While their tissue of origin remains uncertain, it is postulated that low-grade serous OvCa may also originate from the fallopian tubes [1,2].

The most critical non-modifiable risk factors for ovarian cancer are germline mutations in BRCA1 and BRCA2. While fewer than 1% of individuals in the general population carry these mutations, their prevalence among women diagnosed with high-grade serous carcinoma (HGSC) is approximately 20–25%. BRCA1/2 mutations are estimated to account for approximately 25% of all OvCa cases. In contrast, modifiable risk factors—such as obesity, smoking, and physical inactivity—are thought to contribute to approximately 10% of cases. Among modifiable protective factors, breastfeeding has been associated with a reduced risk, potentially preventing 4–5% of OvCa cases [1,2]. Early-stage OvCa can be treated successfully, with surgical resection followed by chemotherapy, achieving a 5-year overall survival (OS) rate of 70–95%. Unfortunately, the majority of patients (approximately 80%) are diagnosed at an advanced stage, at which point the prognosis is significantly poorer. For advanced OvCa, the 5-year OS drops dramatically to between 10% and 40% [2].

At the molecular level, OvCa is characterised by mutations in genes encoding proteins involved in DNA repair processes. The most common molecular aberrations underlying OvCa development are germline mutations in genes responsible for homologous recombination, a key DNA repair mechanism. In particular, BRCA1 and BRCA2 mutations are associated with an estimated 61% and 24% lifetime risk, respectively, of developing OvCa—especially high-grade serous carcinoma (HGSC) [1]. Other affected genes include RAD51C, RAD51D, PALB2, and BRIP1, which also function in homologous recombination, as well as MLH1, MSH2, and MSH6, which are involved in the DNA mismatch repair pathway [1].

The critical role of DNA repair in ovarian cancer (OvCa) is underscored by the clinical success of poly(ADP-ribose) polymerase (PARP) inhibitors, particularly in patients with BRCA-mutated tumours. PARP inhibitors, such as olaparib, have demonstrated a high initial response rate of approximately 80% in this patient population [2]. Standard treatment regimens also include cytoreductive surgery and traditional chemotherapy, primarily based on platinum compounds and taxane derivatives [5]. PARP inhibitors exert their therapeutic effect by inducing synthetic lethality in cancer cells deficient in homologous recombination repair. In normal cells, PARP enzymes are essential for the repair of single-strand DNA breaks (SSBs). Inhibition of PARP leads to its trapping at damaged DNA and, as a consequence, to the accumulation of unrepaired SSBs, which are subsequently converted into double-strand breaks (DSBs) during DNA replication. In healthy cells, these DSBs are efficiently repaired via homologous recombination, a pathway that relies on functional BRCA1 and BRCA2 proteins. However, in OvCa cells harbouring BRCA1/2 mutations, this pathway is impaired, which leads to persistence of DNA damage, genomic instability, and cell death [6,7]. Despite the promising initial responses, resistance to PARP inhibitors remains a major clinical challenge. More than 70% of patients treated with PARP inhibitors experience disease relapse within two years [2].

Human cells express approximately 2600 microRNAs (miRNAs), which are predicted to regulate over 60% of the protein-coding genes [8]. The complexity of this regulatory network is substantial, as a single miRNA can target multiple mRNAs, and conversely, a single gene may be regulated by numerous miRNAs. This redundancy and overlap often complicate the estimation of individual miRNA effects on gene expression. miRNAs are generated through a multistep biogenesis pathway. Transcription of miRNA-coding genes produces primary transcripts (pri-miRNAs), which are subsequently cleaved in the nucleus by the RNase III enzyme Drosha, resulting in precursor miRNAs (pre-miRNAs). These are exported to the cytoplasm via the Exportin-5 (XPO5) transporter. In the cytoplasm, the pre-miRNAs undergo further processing by the endoribonuclease Dicer, producing double-stranded miRNA duplexes. One strand of the duplex is then loaded onto the Argonaute 2 (Ago2) protein to form the RNA-induced silencing complex (RISC). This complex facilitates binding of the mature miRNA to complementary sequences—typically located in the 3′ untranslated region (3′UTR) of the target mRNAs—leading to mRNA degradation or translational repression [8].

The global interest in RNA-based therapeutics has grown significantly, particularly following the success of RNA-based vaccines against SARS-CoV-2. This momentum has extended to miRNA-targeting strategies. Although an increasing number of clinical trials are investigating miRNA-based therapeutics, none have yet received regulatory approval from the FDA or EMA [9].

In this review, we focus on miRNAs that regulate DNA repair pathways in ovarian cancer, as these molecules hold considerable promise as clinically relevant diagnostic and predictive biomarkers, as well as potential therapeutic targets.

## 2. DNA Repair Mechanisms in OvCa: Role of miRNAs

**Figure 1:**
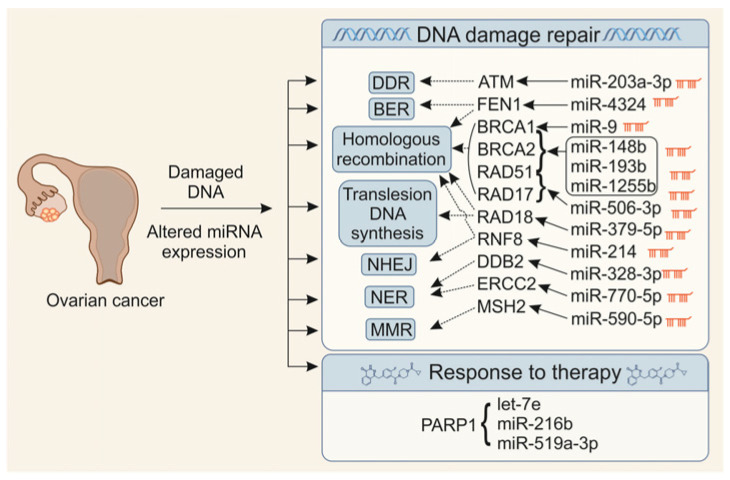
microRNAs targeting DNA damage repair-related genes in ovarian cancer. DDR: DNA damage response; BER: Base excision repair; NHEJ: non-homologous end joining, NER: nucleotide excision repair, MMR: mismatch repair. See text for details.

Ovarian cancer (OvCa) is characterised by alterations in all major DNA damage repair (DDR) mechanisms. These include direct reversal repair (DR), mismatch repair (MMR), nucleotide excision repair (NER), base excision repair (BER), and double-strand break (DSB) repair via both homologous recombination (HR) and non-homologous end joining (NHEJ). In addition, DNA damage response (DDR)-associated processes, such as chromatin remodelling, checkpoint signalling, and the TP53 pathway, are frequently disrupted in OvCa cells (Figure 1). These canonical mechanisms have been comprehensively reviewed in excellent previous articles [10,11]. Therefore, in this review, we focus primarily on miRNA-mediated mechanisms that regulate the expression and function of proteins involved in DNA repair.

The DDR encompasses a network of pathways that detect DNA lesions, activate checkpoint signalling cascades to arrest the cell cycle, mediate repair of damaged DNA, and activate senescence or cell death. It involves proteins that act as sensors of DNA damage, transducers of intracellular signals, and effectors that execute DNA repair. DDR activation enables the recruitment of repair pathways tailored to the type of lesion. Traditionally, DDR is described in the context of cell response to SSBs and DSBs that are resolved by single-strand break repair (SSBR) or DSB repair (DSBR) mechanisms, including classical NHEJ, alternative NHEJ, and single-strand annealing (SSA) [12]. The primary role of the DDR is to maintain genomic integrity, thereby ensuring accurate DNA replication and cell viability. Importantly, OvCa is characterised by dysregulated expression of multiple miRNAs that modulate the levels and activity of proteins across DDR and all of the DNA repair pathways.

### 2.1. miRNAs Targeting Transcripts of DNA Repair-Related Genes: Roles in OvCa Tumorigenesis

Dysregulated expression of miRNAs targeting genes involved in DNA repair may directly contribute to ovarian cancer (OvCa) tumorigenesis (Table 1). In particular, several tumour-suppressive miRNAs are downregulated in OvCa, resulting in the elevated expression of their oncogenic targets.

ATM (Ataxia Telangiectasia Mutated), a serine/threonine protein kinase, plays a central role in the DNA damage response (DDR), especially in the response to double-strand breaks (DSBs). ATM-mediated phosphorylation of key DSB repair proteins facilitates their interaction and promotes the assembly of large multiprotein repair complexes [69]. Elevated ATM expression has been observed in OvCa tumours and is associated with poor patient survival. This overexpression coincides with the downregulation of miR-203a-3p, which has been identified as a direct regulator of ATM. Notably, miR-203a-3p reduces OvCa cell proliferation and promotes apoptosis, and its effects on migration and invasion are mimicked by silencing ATM [23]. This suggests that at least some tumour-suppressive effects of miR-203a-3p may be potentially mediated through ATM targeting.

Another key DDR gene is CHEK2 (Checkpoint Kinase 2), encoding a serine/threonine kinase, which is a downstream target of ATM. Upon phosphorylation by ATM, CHEK2 activates multiple substrates, including those involved in cell cycle arrest. This, in turn, allows time for DNA repair prior to progression through the cell cycle. Owing to its role in inhibiting uncontrolled proliferation and supporting genomic stability, CHEK2 is considered a tumour suppressor. In osteosarcoma and breast cancer cells, CHEK2 is targeted by several miRNAs, including miR-191 [26] and miR-182-5p [28], respectively. Both miR-191 [27,70] and miR-182-5p [30] are upregulated in the serum or tumour tissue of OvCa patients. However, the functional impact of these miRNAs on CHEK2 expression in OvCa cells remains to be experimentally validated. Interestingly, genomic variation in the miR-191 sequence has been suggested to predispose to familial ovarian cancer in patients without the BRCA1/2 or MMR gene mutation background [71]. Moreover, miR-191 also targets a binding site, which is created by SNP34091 located in the 3′UTR of MDMX (also known as MDM4), leading to decreased MDMX expression. MDMX is a nuclear protein that inhibits the tumour suppressor p53, thereby promoting cancer progression. In OvCa patients, the presence of the wild-type MDMX allele (lacking the miR-191 binding site) has been linked to increased MDMX mRNA and protein levels. Moreover, this allele is also associated with increased recurrence and poorer survival among oestrogen receptor (ER)-negative patients [59]. MDMX, as well as MDM2, another gene involved in p53 regulation, are also targeted by miR-214-5p in immature ovarian teratocarcinoma (IOT). MDM2 encodes an E3 ubiquitin ligase responsible for p53 ubiquitination and subsequent proteasomal degradation. In IOT, miR-214-5p expression is reduced due to sequestration by the long non-coding RNA LINC00324, resulting in upregulation of p53-inhibitory proteins and contributing to enhanced cell proliferation and reduced apoptosis [60]. Another miR targeting MDM2 in OvCa is miR-647, which also constitutes part of a regulatory loop involving circ-FAM53B-mediated miR-647 sponging [58]. Moreover, in OvCa, in general, miR-214 is upregulated [72] and targets RNF8, another ubiquitin ligase that is recruited to DSBs after ATM-mediated MDC1 (Mediator of DNA Damage Checkpoint-1) phosphorylation. This in turn initiates a cascade of ubiquitination events leading to the assembly of downstream repair machinery, including 53BP1 (thus intimating NHEJ) or BRCA1 (thus initiating HR). miR-214-mediated RNF8 targeting and downregulation induce genomic instability in OvCa cells [34]. Importantly, in OvCa, p53, a master regulator of cell fate in response to DNA damage, is regulated by several miRNAs, including miR-488 [46] and miR-1228 [52]. Conversely, p53 regulates transcription of the miR-200 family and the miR-34 family’s miRNAs, as well as miR-31 and miR-145 in OvCa [40,45,52,57]. miR-34 miRNAs are directed to multiple targets controlling cell cycle and apoptosis, including p53 itself, and are repressed in OvCa due to *TP53* mutation prevalence. Low miR-34 miRNA expression correlates with poor OvCa prognosis [40,41].

MBD4 (Methyl-CpG Binding Domain Protein 4, also known as MED1) is a mismatch-specific DNA N-glycosylase involved in base excision repair (BER). It acts as a thymine and uracil glycosylase, specifically repairing G:T mismatches that arise from the spontaneous deamination of 5′-methylcytosine (5mC). Germline mutations in MBD4 are associated with a multi-tumour predisposition syndrome, which includes ovarian granulosa cell tumours [73]. Network-based analyses have predicted MBD4 as a potential target of miR-196a-2. Notably, the miR-196a-2 rs11614913 single-nucleotide polymorphism (SNP) is observed with a higher frequency in patients with HGSC compared with healthy controls. miR-196a expression itself is upregulated in ovarian cancer [13,14]. However, to date, no studies have directly evaluated MBD4 expression in OvCa. Our unpublished analysis of TCGA data indicates reduced MBD4 expression in OvCa tumours, potentially reflecting the upregulation of miR-196a. Nevertheless, experimental validation is required to confirm whether MBD4 is directly targeted by miR-196a in ovarian cancer cells.

FEN1 (Flap Structure-Specific Endonuclease 1) is involved in both DNA replication and repair. It plays a critical role in the rapid repair of SSBs [74], as well as in homologous recombination (HR) [75]. FEN1 is overexpressed in OvCa tumours, where it contributes to tumorigenesis by promoting proliferation, migration, colony formation, and reducing cell adhesion. It is a direct target of miR-4324, whose expression is significantly downregulated in OvCa, thereby enabling oncogenic FEN1 activity and tumour growth in vivo [16]. In addition to miR-4324, miR-134-3p has also been reported to exert tumour-suppressive effects by directly targeting FEN1. It induces apoptosis and cell cycle arrest while inhibiting proliferation, migration, and invasion of OvCa cells in vitro [17]. Other miRNAs, such as miR-140 and miR-193b, have been shown to regulate FEN1 in breast cancer and osteosarcoma [76,77], although their specific role in modulating FEN1’s DNA repair functions in OvCa remains unknown.

OvCa tumours also aberrantly overexpress miR-210, a well-known oncogenic miRNA that promotes epithelial-to-mesenchymal transition (EMT) and tumour growth. In breast cancer, miR-210 has been shown to target RAD52, a key mediator of HR and SSA [19,64,65]. In SSA, RAD52 initiates the repair by mediating pairing of the homologous sequences, whereas in HR, it facilitates DNA strand exchange by binding to and activating RAD51, a central recombinase in HR [78,79]. Whether miR-210 exerts similar pro-tumourigenic effects in OvCa by suppressing RAD52 remains to be determined.

DDB2 (Damage-Specific DNA Binding Protein 2) is a crucial component of the UV-DDB complex, which recognises DNA lesions induced by ultraviolet light and recruits nucleotide excision repair (NER) machinery [80,81]. In ovarian cancer, DDB2 acts as a tumour suppressor by reducing the self-renewal capacity of OvCa stem cells [82]. Consistent with this function, reduced DDB2 expression is associated with poor prognosis in OvCa patients [82,83]. In OvCa cells, DDB2 is targeted and downregulated by miR-328-3p, which impairs its tumour-suppressive activity. Remarkably, DDB2 also suppresses the expression of ALDH1A1 (Aldehyde Dehydrogenase 1 Family Member A1), a cytosolic dehydrogenase involved in retinoic acid metabolism, lipid peroxidation, and fructosamine degradation [83,84,85,86]. ALDH1A1 is a well-established promoter of oncogenesis in ovarian cancer, and its inhibition has been shown to induce necroptosis in OvCa stem cells [87]. Furthermore, pharmacological inhibition of the ALDH1A family not only induces DNA damage in OvCa cells but also sensitises them to inhibitors targeting ATM or ATR, key regulators of the DNA damage response [88].

### 2.2. miRNAs Targeting Transcripts of DNA Repair-Related Genes: Roles in OvCa Therapy and Chemoresistance

Multiple in vitro and preclinical in vivo studies have demonstrated that miRNAs associated with DNA repair play a significant role in modulating OvCa response to therapeutic interventions, contributing to both chemotherapy resistance and sensitivity (Table 2). Notably, drugs based on some of these miRNAs, such as miR-21 and miR-155, have already been evaluated in clinical trials, although in cancers other than OvCa [8]. miR-21 and miR-155 are pleiotropic regulators that modulate the expression of numerous genes, including but not limited to those involved in DNA repair. Therefore, any potential clinical benefits associated with miR-21 or miR-155-targeted therapeutics may not necessarily arise from their effects on DNA repair pathways. To the best of our knowledge, none of the miRNAs discussed in this review has yet been evaluated in clinical trials specifically in the context of OvCa. This underscores the need for further research and robust clinical validation before the encouraging findings from preclinical studies can be translated into clinical applications.

#### 2.2.1. miRNAs Targeting PARP1

PARP1 (Poly(ADP-Ribose) Polymerase 1) encodes a poly(ADP-ribosyl)transferase that catalyses the post-translational modification of proteins involved in DNA repair. During the PARP1-mediated reaction, an ADP-D-ribosyl group is transferred from NAD^+^ to the carboxyl group of target amino acid residues. Subsequent elongation involves the addition of ADP-ribosyl units to the 2′-position of the terminal adenosine, forming poly(ADP-ribose) (PAR) chains typically consisting of 20–30 units.

PARP1 has emerged as a central therapeutic target in OvCa, particularly in patients harbouring BRCA mutations. In the context of DNA repair, PARP1 detects and binds to SSBs, triggering its auto-PARylation. This modification facilitates the recruitment of key DNA repair proteins, including XRCC1 (a scaffold protein), DNA polymerase β (Polβ), and DNA ligase III (LigIII), thereby enabling the efficient repair of DNA lesions [132]. Due to its central role as a hub protein recruiting the crucial components of DNA mechanisms, PARP1 is involved in multiple DNA repair pathways, including BER, NER, MMR, HR, or NHEJ [133]. Inhibition of PARP1 leads to the accumulation of unrepaired SSBs, which are converted into double-strand breaks (DSBs) during DNA replication. In cells deficient in DSB repair mechanisms, such as those with BRCA1/2 mutations, this accumulation of DNA damage ultimately results in cell death via synthetic lethality [134].

PARP1 is considered an oncogenic protein in ovarian cancer. Elevated PARP1 expression is associated with poor patient survival and contributes to increased cell viability, migration, invasion, and tube formation in OvCa cells. Notably, PARP1 is directly targeted by miR-519a-3p, whose expression is downregulated in OvCa tumours. This reduction facilitates upregulation of PARP1, thereby enhancing its oncogenic functions [15]. The exceptional clinical response of BRCA-mutated OvCa patients to PARP1 inhibition led to the approval of the first PARP1 inhibitor, olaparib, by the FDA in 2014, followed by niraparib in 2020 [135]. However, a substantial proportion of patients eventually develop resistance to PARP1 inhibitors. This necessitates the development of complementary strategies to suppress PARP1 activity. In this context, miRNAs that regulate PARP1 expression or interfere with resistance to PARP1 inhibitors are of particular interest. For instance, Xiao et al. demonstrated that reduced expression of let-7e in OvCa contributes to cisplatin resistance via loss of PARP1 suppression. Their study showed that let-7e directly targets and downregulates PARP1, thereby impairing the repair of both SSBs and DSBs [90]. Similarly, miR-216b, which is also downregulated in cisplatin-resistant OvCa tumours, targets PARP1. Restoration of miR-216b expression reduces PARP1 levels and reverses cisplatin resistance. Furthermore, reintroduction of miR-216b into OvCa cells suppresses tumour formation in nude mouse models [91]. Moreover, miR-622 was found to promote homologous recombination (HR) by targeting Ku70/Ku80, key components of the non-homologous end joining (NHEJ) pathway. Consequently, miR-622-mediated HR restoration reduces sensitivity to PARP1 inhibitors in BRCA1-mutated ovarian cancer (OvCa) cells [111]. However, the clinical and translational relevance of these findings remains limited due to significant technical challenges that must be addressed prior to evaluating these specific miRNAs in clinical trials involving OvCa patients (further discussed in Section 4).

#### 2.2.2. miRNAs Targeting Other Genes Involved in DNA Repair

The most critical genetically determined risk factors for ovarian cancer (OvCa) are germline BRCA1 and BRCA2 mutations, which result in dysfunction of DNA repair mechanisms. BRCA1 (BRCA1 DNA Repair Associated, also known as Breast Cancer Type 1 Susceptibility Protein) and BRCA2 (BRCA2 DNA Repair Associated, or Breast Cancer Type 2 Susceptibility Protein) encode proteins that function as E3 ubiquitin ligases. These proteins interact with and recruit multiple components of the homologous recombination (HR) and Fanconi anaemia pathways, supporting DNA end resection and protecting nascent DNA strands during repair [136,137]. Loss-of-function mutations in BRCA1/2 have profound consequences for DNA repair fidelity. The absence of functional BRCA proteins impairs HR, leading to a state known as homologous recombination deficiency (HRD). In this state, the cells are forced to rely on error-prone repair mechanisms, such as non-homologous end joining (NHEJ). This shift promotes genomic instability, facilitates the accumulation of mutations, and may activate oncogenic pathways [138,139]. Paradoxically, while BRCA1/2 mutations increase cancer risk, they also enhance tumour sensitivity to DNA-damaging therapies. This is due to synthetic lethality—whereby BRCA-deficient cells are unable to effectively repair therapy-induced DNA damage, resulting in cell death. Consequently, miRNAs that suppress BRCA1/2 expression may contribute to improved treatment responses. Indeed, miR-9 has been shown to bind directly to the 3′ untranslated region (3′UTR) of BRCA1 mRNA, downregulating its expression in OvCa cells and enhancing cisplatin sensitivity in in vivo models. Consistent with these findings, high miR-9 expression in OvCa tumours correlates with improved chemotherapy response and prolonged progression-free survival [120]. Interestingly, deletion of miR-1255b, miR-193b and miR-148b, which target BRCA1, BRCA2 and RAD51, the central HR recombinase, in OvCa tissues results in activation of HR in G1 phase of the cell cycle, where it should be normally suppressed. This in turn leads to an increased number of genomic copy alterations [63]. miRNAs can also sensitise OvCa cells to treatment by targeting genes beyond PARP1 and BRCA1, including RAD18 (RAD18 E3 Ubiquitin Protein Ligase), a key regulator of translesion DNA synthesis (TLS) and homologous recombination (HR) repair [140]. RAD18 functions as an E3 ubiquitin-protein ligase that forms a complex with UBE2B (an E2 ubiquitin-conjugating enzyme) to catalyse the mono-ubiquitination of PCNA (Proliferating Cell Nuclear Antigen). This modification facilitates the recruitment of downstream DNA repair proteins [141,142]. Notably, both RAD18 and UBE2B have been shown to play oncogenic roles in OvCa [142]. RAD18 is a predicted target of several miRNAs, including miR-145, miR-379-5p, and miR-630, though not all of these interactions have been experimentally confirmed in OvCa cells [93,94,95,96,97,98,99,100,101]. Among these, miR-379-5p appears to have the greatest clinical relevance. It has been demonstrated to inhibit stemness-associated properties in OvCa stem cells and to enhance their sensitivity to cisplatin treatment [93]. These effects closely mirror those observed following RAD18 silencing in OvCa stem cells. Moreover, miR-379-5p-mediated downregulation of RAD18 expression impairs DNA repair in ovarian cancer (OvCa) stem cells. Mechanistically, miR-379-5p directly targets RAD18, thereby preventing the mono-ubiquitination of PCNA. This modification is essential for the recruitment of translesion synthesis (TLS) polymerases to sites of DNA damage. As a result, disruption of PCNA mono-ubiquitination impairs the lesion bypass function of polymerase eta (POLH/RAD30A; Polη), leading to the accumulation of unrepaired DNA damage and ultimately triggering apoptosis in OvCa cancer stem cells (CSCs) [93].

miR-211 targets several DNA repair genes, including POLH, a specialised TLS DNA polymerase involved in UV-induced lesion bypass, TDP1, a tyrosyl DNA phosphodiesterase that is an end processor implicated in SSBR and DSBR, ATRX, a chromatin remodeler, MRPS11, a mitochondrial ribosomal protein, and ERCC6L2, an NHEJ helicase in human embryonic kidney cells. Higher miR-211 levels allow for better prediction of response in OvCa patients to platinum compounds [89].

miR-506-3p targets RAD51, a DNA recombinase essential for homologous recombination (HR), where it promotes strand invasion and exchange between homologous DNA sequences [79]. In addition to its role in HR, RAD51 also supports mitotic DNA synthesis and ensures accurate chromosome segregation during mitosis [143]. Increased expression of miR-506-3p in ovarian cancer (OvCa) tumours correlates with improved responses to platinum-based chemotherapy, as well as prolonged progression-free survival (PFS) and overall survival (OS). It has been demonstrated that miR-506-3p directly targets and reduces RAD51 expression, thereby impairing HR efficiency. This results in elevated DNA damage and sensitisation of OvCa cells to cisplatin and olaparib in in vivo models [128]. Interestingly, miR-506-3p also targets RAD17, another critical HR-related gene. RAD17 functions as a sensor in the DNA damage checkpoint pathway and facilitates recruitment of the MRE11-RAD50-NBS1 (MRN) complex during HR repair [144]. Inhibition of RAD17 by miR-506-3p enhances platinum sensitivity and induces synthetic lethality when combined with inhibitors of the cell cycle checkpoint kinases CHEK1 and WEE1. These effects have been observed in OvCa cells that were otherwise resistant to cisplatin treatment [129].

Ataxia telangiectasia and Rad3-related kinase (ATR) and checkpoint kinase 1 (CHEK1) are the key components of the DNA damage response (DDR). They play essential roles in sensing SSBs and coordinating cell cycle arrest to facilitate repair. Several studies have demonstrated that inhibition of ATR and CHEK1 can synergize with PARP1 inhibition, yielding enhanced therapeutic effects in preclinical OvCa models [145,146]. It is plausible that some of these synergistic interactions are mediated, at least in part, by miRNAs. For example, combined treatment of OvCa cells with the PARP1 inhibitor olaparib and the ATR inhibitor ceralasertib resulted in downregulation of several miRNAs, including miR-100-5p, miR-26a-5p, miR-33a-3p, miR-99b-5p, and miR-486-5p—changes not observed when either agent was administered alone. Additionally, combined treatment with olaparib and the CHEK1 inhibitor MK-8776 led to the upregulation of miR-1290 [92]. These findings suggest that dual inhibition strategies can reshape miRNA expression profiles, potentially contributing to therapeutic efficacy. Furthermore, treatment of OvCa cells with KU60019, an inhibitor of ATM kinase, resulted in the downregulation of miR-1273g-3p. This miRNA targets DGAT1 (Diacylglycerol O-Acyltransferase 1), a gene involved in lipid metabolism. Downregulation of miR-1273g-3p releases its inhibitory effect on DGAT1, leading to increased expression. Interestingly, pharmacological inhibition of DGAT1 alleviated KU60019-induced apoptosis. This suggests that miRNA-mediated suppression of DGAT1 may indeed contribute to the cytotoxicity induced by ATM inhibition [147].

Interestingly, several miRNAs are implicated in modulating chemotherapy-induced DNA damage, although the specific gene targets mediating these effects remain largely unidentified. One such example is miR-125a-3p. Combined treatment of ovarian cancer (OvCa) cells with olaparib and cisplatin has been shown to increase miR-125a-3p expression. Overexpression of miR-125a-3p in OvCa cells leads to elevated levels of DNA damage, as indicated by increased accumulation of γ-H2AX, a phosphorylated form of histone H2AX that is rapidly induced in response to DNA damage, particularly double-strand breaks (DSBs) [102]. In the same study, BOK (BCL2-related ovarian killer) was predicted to be modulated by miR-125a-3p. However, BOK is an apoptosis regulator and has not been directly associated with regulating DNA damage repair. This implies that the DNA damage-promoting effects of miR-125a-3p may be mediated through alternative, as yet unidentified, possibly several targets [102].

ERCC2 (ERCC Excision Repair 2, TFIIH Core Complex Helicase Subunit) is an ATP-dependent 5′–3′ DNA helicase involved in the nucleotide excision repair (NER) pathway. Expression of miR-770-5p is reduced in OvCa tumours resistant to chemotherapy, and low miR-770-5p levels correlate with poor patient survival. Overexpression of miR-770-5p enhances cisplatin sensitivity in OvCa cells. These effects are mediated by ERCC2, a direct target of miR-770-5p, as demonstrated by ERCC2 silencing. The latter replicated the increased cisplatin sensitivity observed with miR-770-5p overexpression. Furthermore, either increased miR-770-5p expression or decreased ERCC2 levels impaired the repair of DNA damage induced by cisplatin treatment [103].

Cisplatin resistance in OvCa is also modulated by several miRNAs that regulate the expression of genes involved in the MMR pathway. miR-590-5p has been shown to directly target and downregulate MSH2 (MutS Homolog 2), a core component of the MMR system. MSH2 forms heterodimers with MSH6 or MSH3 to generate complexes that recognise mismatched DNA, bend the DNA helix, and recruit downstream repair factors [148]. In cisplatin-resistant OvCa cells, MSH2 expression is reduced, while its restoration re-sensitizes cells to cisplatin-induced cytotoxicity. Conversely, miR-590-5p is upregulated in cisplatin-resistant OvCa and suppresses MSH2 expression [105]. However, the aforementioned study did not assess the functional consequences of miR-590-5p on MSH2-mediated MMR. On the other hand, MSH2, along with other MMR genes such as MSH6 and MLH1, is a known target of miR-155 in colorectal cancer, where miR-155-mediated suppression leads to microsatellite instability. miR-155 expression is also elevated in OvCa tumours compared with normal ovarian tissues, and in metastatic versus localised disease [22]. However, that study did not explore whether MSH2 or other MMR genes are direct targets of miR-155 in OvCa, leaving its role in MMR suppression in this context unresolved. Similarly, MSH2 and MSH6 have been reported as targets of oncogenic miR-21 in colorectal cancer, contributing to reduced sensitivity to 5-fluorouracil (5-FU) [106]. In OvCa, miR-21 is also significantly upregulated and has been implicated in mediating cisplatin resistance [107]. It remains unclear whether this resistance involves suppression of MMR proteins. Given that miR-21 targets several key oncogenic regulators in OvCa—including PTEN, PDCD4, and CDK6 [107]—it is likely that its contribution to drug resistance and tumour progression is mediated through multiple pathways, with MMR playing a less central role.

Paclitaxel resistance in OvCa might be mediated by miRNAs targeting DNA repair genes, including miR-630 that targets RAD18 and PANCG in liver cancer cells, miR-194-5p targeting MDM2, and miR-185 targeting ATR in renal cancer cells [99,115,119]. Nevertheless, involvement of these miRNA-DNA repair gene interactions in paclitaxel resistance necessitates empirical confirmation with the use of OvCa cells.

A number of other miRNAs that are dysregulated in OvCa have been shown to target DNA repair genes in other cancer types. For example, miR-449a has been reported to target EME1 and downregulate BRCA2 and RAD51 in breast cancer (Table 2), while miR-146 targets and/or downregulates FANCM and BRCA1 in cervical, gastric, and breast cancer cells (Table 1). However, all of these miRNA–target interactions require experimental validation in the context of OvCa.

## 3. miRNAs Regulated by Proteins Involved in DNA Damage Repair

miRNAs not only regulate the expression of genes involved in DNA repair signalling pathways, but their own expression can also be modulated by DNA repair-related genes. DDX1 is an ATP-dependent RNA helicase that facilitates the unwinding of RNA–RNA and RNA–DNA duplexes. Intriguingly, DDX1 has been identified as a regulator of miRNA expression following DNA damage in OvCa cells. Specifically, DDX1 facilitates the recruitment of primary miRNA transcripts (pri-miRNAs) to Drosha, the catalytic subunit of the Microprocessor complex responsible for the nuclear cleavage of pri-miRNAs into precursor miRNAs (pre-miRNAs). As a result, DDX1 promotes the maturation and expression of multiple miRNAs, particularly members of the miR-200 family, which are known to regulate epithelial–mesenchymal transition (EMT). Upon DNA damage, DDX1 is phosphorylated by ATM kinase and recruited to sites of DNA lesions. In OvCa, the expression of miR-200 family members is reduced, and DDX1 functions as a tumour suppressor by promoting miR-200 expression and thereby inhibiting EMT, invasion, and metastasis [24]. It is also known that miRNA biogenesis is regulated by other DNA repair proteins. For example, CtIP (C-terminal-binding protein-interacting protein), which plays a role in the processing of DNA double-strand breaks (DSBs) during homologous recombination, has been shown to modulate miRNA expression [149]. Similarly, BRCA1 has been implicated in miRNA regulation [150]. Whether these proteins influence miRNA expression in OvCa remains to be elucidated.

## 4. Limitations and Challenges

The promising results of in vitro and preclinical studies demonstrating the diagnostic and therapeutic potential of miRNAs have prompted numerous clinical trials investigating their efficacy in the detection and treatment of both cancerous and non-cancerous diseases. At the time of writing this review, a search of ClinicalTrials.gov using the terms ‘ovarian cancer’ and ‘microRNA’ yielded 22 results (https://clinicaltrials.gov/search?cond=Ovarian%20Cancer&term=microRNA, accessed on 9 September 2025). However, only 16 of these studies are specifically focused on malignant disease, while the remaining trials pertain to non-cancerous conditions, such as polycystic ovary syndrome (PCOS). Furthermore, the majority of these studies investigate microRNAs as potential biomarkers, with none exploring their use as therapeutic agents. Likewise, none of the miRNA-based drugs tested in the treatment of various diseases has yet received approval from the US Food and Drug Administration (FDA) [8,151]. The clinical translation of miRNAs as therapeutic agents faces several significant challenges that currently limit their utility. A key limitation arises from the incomplete understanding of the full spectrum of genes regulated by individual miRNAs. Each miRNA typically targets multiple genes involved in diverse cellular pathways, and this broad regulatory scope increases the likelihood of off-target effects and associated toxicity—one of the most critical obstacles in miRNA-based therapy [8]. Even when a potential miRNA–mRNA interaction is predicted computationally based on sequence complementarity, such interactions must be rigorously validated in in vivo systems to confirm their biological relevance. miRNA-based therapeutics are typically designed around two main strategies: miRNA mimics, which are used to restore the function of downregulated tumour-suppressive miRNAs, and antagomirs, which are chemically modified oligonucleotides designed to inhibit overexpressed oncogenic miRNAs [151]. However, the clinical application of both approaches is hindered by common technical barriers. They include inefficient delivery to target tissues and the risk of immune activation or toxicity associated with the introduced oligonucleotides [151]. Additional challenges specific to ovarian cancer stem from the molecular characteristics of the disease. OvCa is marked by significant intra-tumoural and inter-tumoural heterogeneity, which can influence both miRNA expression and function [152]. This heterogeneity complicates the development of standardised miRNA-based therapies that would be universally effective across patients. Many initially promising clinical trials testing the efficacy of miRNA-based therapeutics have been discontinued due to severe adverse effects, underscoring the need for improved delivery systems, enhanced targeting specificity, and a deeper understanding of miRNA regulatory networks in the context of OvCa.

The perspectives of miRNAs as diagnostic tools are much more promising. Although no miRNA-based diagnostic tools have yet been approved specifically for OvCa diagnosis or monitoring, several clinically validated and/or commercially available miRNA-based tests are already implemented in hospital settings. One notable example is GASTROClear™, which has been approved by the Singapore Health Sciences Authority and received the FDA Breakthrough Device Designation in the United States. This test employs qPCR-based analysis of 12 circulating blood miRNAs for the early detection of gastric cancer in high-risk individuals (https://mirxes.com/mirxes-receives-fdas-breakthrough-device-designation-for-gastroclear-to-advance-blood-based-cancer-early-detection; accessed 6 September 2025).

Other miRNA-based diagnostic assays still await regulatory approval. These include the hepato-miR^®^ Kit, designed to assess the risk of post-hepatectomy liver failure (https://www.tamirna.com/hepatomir-kit-ce-ivd/; accessed 6 September 2025), and the miR Sentinel™ Prostate Cancer Test, which analyses urine-derived miRNAs for prostate cancer detection (https://www.mirnascientific.com; accessed 6 September 2025).

As with all biomarkers, the clinical utility of circulating miRNAs depends on several critical factors. These include miRNA stability, specificity, and the standardisation of analytical workflows. miRNAs detectable in biofluids, such as serum, plasma, urine, or saliva, exhibit remarkable stability, largely attributed to their encapsulation within extracellular vesicles (e.g., exosomes) or their association with stabilising proteins, such as Argonaute 2 (Ago2). miRNAs are generally considered highly stable molecules; for instance, deep-frozen miRNAs can remain intact for up to two years. Nevertheless, some studies have raised concerns regarding the impact of repeated freeze–thaw cycles on miRNA integrity in serum samples [153].

Technical challenges in miRNA biomarker analysis primarily stem from the lack of standardisation across all procedural steps, including biofluid collection, sample storage, RNA isolation, and quantification methods. These inconsistencies can significantly affect the reproducibility and reliability of miRNA measurements [153]. Another major limitation is the low disease specificity of many miRNAs. Although numerous studies have reported altered expression of specific miRNAs—such as miR-21—in various cancers, these changes are often observed across multiple malignant and benign conditions. Consequently, such miRNAs may reflect general pathological processes rather than indicate a specific disease entity [154].

## 5. Conclusions

Ovarian cancer (OvCa) is one of the tumour types most strongly associated with dysfunction in DNA repair pathways. This association is particularly evident in hereditary OvCa cases, which frequently result from germline mutations in BRCA1 or BRCA2. miRNAs constitute a critical component of the molecular landscape of OvCa, contributing to tumour initiation, progression, and resistance to therapy. At least some of these effects are mediated through direct regulation of DNA repair genes, which serve as validated miRNA targets—for example, miR-203a-3p targeting ATM and miR-519a-3p targeting PARP1. The number of miRNAs involved in DNA repair dysfunction in OvCa may be substantially higher, pending experimental validation of predicted interactions—such as miR-196a with MBD4, or miR-191 and miR-182-5p with CHEK2. Similarly, the mechanistic targets of miRNAs that have been shown to influence DNA damage but lack confirmed gene targets—such as miR-125a-3p—should be systematically identified. Crucially, the functional relevance of miRNA–target interactions must be examined in both in vitro and in vivo models to determine their true impact on the efficacy of DNA repair processes.

In conclusion, miRNAs that regulate DNA repair genes represent promising candidates for future clinical applications in OvCa, both as diagnostic and predictive biomarkers and as therapeutic targets. However, further research is essential to develop safe, effective, and targeted miRNA-based therapeutics or biomarkers tailored specifically to the complex biology of ovarian cancer.

## Figures and Tables

**Table 1:** miRNAs targeting genes involved in DNA repair that contribute to OvCa tumorigenesis and/or represent potential OvCa biomarkers.

miRNA	Targeted Gene/Protein(s)	Related DNA Repair Pathway(s)	Effect	miRNA-Target Gene Interaction Validation	References
miR-196a-2	MBD4 (Methyl-CpG Binding Domain-4, DNA Glycosylase)	Base excision repair	miR-196a-2 rs11614913 SNP is frequent in high-grade serous OvCa. Network analysis predicted MBD4 as its target. miR-196a is upregulated in OvCa.	Not validated—based on network analysis.	[13,14]
miR-519a-3p	PARP1 (Poly(ADP-Ribose) Polymerase-1)	Mainly single-strand break repair, but also other repair pathways	Downregulated in OvCa. Targets PARP1 to inhibit malignant traits of OvCa cells.	Validated—dual luciferase reporter and biotinylated miRNA pull-down assays in OvCa cells.	[15]
miR-43 2 4	FEN1 (Flap Structure-Specific Endonuclease-1)	Single-strand break repair, homologous recombination	Downregulated in OvCa tissues and cells. Targets FEN1 to repress OvCa cell growth and cancer progression.	Validated—dual luciferase reporter assay in OvCa cells.	[16]
miR-13 4-3 p			Downregulated in OvCa tissues and cells. Targets FEN1 to inhibit malignant traits of OvCa cells.	Validated—dual luciferase reporter assay in OvCa cells.	[17]
miR-328-3 p	DDB2/XPE (Damage Specific DNA Binding Protein-2)	Nucleotide excision repair	Highly expressed in OvCa stem cells. Targets and thus downregulates DDB2 to support stemness phenotype.	Validated—dual luciferase reporter assay in OvCa cells.	[18]
miR-373	RAD23B (UV excision repair protein RAD23 homolog-B); RAD52/RDM1 (RAD52 Homolog, DNA Repair Protein)	Nucleotide excision repair; homologous recombination	Upregulated by HIF1α and targets RAD23B and RAD52 in breast cancer cells. Elevated in OvCa patient’s serum.	Validated—dual luciferase reporter assay in breast cancer cells.	[19,20]
miR-155	MSH2 (MutS Homolog-2), MSH6(MutS Homolog-6), MLH1(MutL Homolog-1)	Mismatch repair	Downregulates MSH2, MSH6 and MLH1, thus inducing microsatellite instability in colorectal cancer. Overexpressed in OvCa tissues and correlates with malignant potential.	Validated—dual luciferase reporter assay in colorectal cancer cells.	[21,22]
miR-203a-3p	ATM (Ataxia Telangiectasia Mutated)	DNA damage response	Downregulated in OvCa tissues. Targets ATM to compromise malignant traits of OvCa cells.	Validated—dual luciferase reporter assay in OvCa cells.	[23]
miR-200a, miR-200b, miR-200c, miR-141, miR-429, miR-200a, miR-29c, miR-141 and miR-101	DDX1 (DEAD-Box Helicase 1)	Clearance of RNA at DNA double-strand breaks	DDX1 regulates post-transcriptional processing and induction of several miRNAs after DNA damage in OvCa cells.	Not applicable.	[24]
miR-1260b	CHK2/CHEK2 (Checkpoint Kinase-2)	DNA damage response	Increased in OvCa serum samples. Predicted to target CHEK2.	Not validated—based on TargetScan, followed by String analysis prediction.	[25]
miR-191			Targets CHEK2 to promote osteosarcoma cell proliferation. Upregulated in ovarian clear cell carcinoma serum samples and suggested as a diagnostic marker.	Validated—dual luciferase reporter assay in osteosarcoma cells.	[26,27]
miR-182-5p	CHK2/CHEK2 (Checkpoint Kinase-2); BRCA1/FANCS(DNA Repair Associated/Breast Cancer Type 1 Susceptibility Protein); RAD17 (Checkpoint Clamp Loader Component)	DNA damage response; homologous recombination; Fanconi anaemia pathway	Targets CHEK2, RAD17 and TP53BP1 in breast cancer cells. Also targets BRCA1 in HeLa cells. Upregulated in OvCa tissue samples.	Validated—dual luciferase reporter assay for CHEK2, RAD17 and TP53BP1 in breast cancer cells. Dual luciferase reporter assay and RNA immunoprecipitation for BRCA1 in HeLa cells.	[28,29,30]
miR-134	H2AX (H2A Histone Family Member X)	DNA damage response	Induced by DNA damage in OvCa cells. Facilitates accumulation of phosphorylated H2AX (γH2AX) and stimulates DNA repair via NHEJ. Promotes OvCa cell survival and xenograft tumour growth in murine model. Also upregulated in OvCa tissues and correlates with poor prognosis.	Interaction with SDS22, regulatory subunit of serine/threonine phosphatase required for mitosis, verified using dual luciferase reporter assay in OvCa cells.	[31]
miR-138			Targets and represses H2AX expression, inhibits HR and triggers chromosomal instability after DNA damage in osteosarcoma cells. Downregulated in invasive OvCa cells. Low expression in patient tissues is correlated with malignant phenotype.	Validated—dual luciferase reporter assay in osteosarcoma cells.	[32,33]
miR-214	RNF8 (Ring Finger Protein-8)	DNA damage response	Targets RNF8, thus suppressing DDR and contributing to chromosomal instability in OvCa cells.	Validated—dual luciferase reporter assay in OvCa cells.	[34]
miR-22	MDC1 (Mediator of DNA Damage Checkpoint-1)	DNA damage response	Targets MDC1 and thus inhibits DNA Repair and triggers genomic instability in several cancer cell models. Targets also TIP-60 in breast cancer cells, and its high expression correlates with metastatic potential. Downregulated in OvCa and low levels correlated with poor prognosis.	Validated—dual luciferase reporter assay for MDC1 in human embryonic kidney cells and for TIP60 in breast cancer cells.	[35,36,37]
miR-34	*TP53*/*p53* (Tumour Protein P53)	DNA damage response; cell cycle checkpoints; apoptosis	TP53 induces miR-34 family after DNA damage. In OvCa, frequent TP53 mutations reduce miR-34a/b/c expression. miR-34s act as tumour suppressors in OvCa, with low levels linked to poor outcome. They also enhance p53 by targeting SIRT1 and MDMX.	Validated—dual luciferase reporter assay for miR-34a and MDMX in colorectal cancer cells, and miR-34a and SIRT1 in human embryonic kidney cells.	[38,39,40,41,42,43,44]
miR-31			Regulated by TP53 and suppressed in TP53-mutated OvCa cells. Predicted to interact with multiple targets among DDR and cell cycle regulatory genes, and higher levels correlated with better prognosis.	Interaction not validated. Predicted from global expression profiling after miR-31 mimic.	[45]
miR-488			Promotes TP53 expression.	Interaction not validated. TP53 upregulated by miR-488 mimic.	[46]
mi R - 29a/b/c			Upregulate TP53 and induce p53-dependent apoptosis through targeting negative p53 regulators p85 alpha and CDC42. Downregulated in OvCa.	Validated—dual luciferase reporter assay for miR-29a/b/c and p85 alpha or *CDC42* in HeLa cells.	[47,48,49]
miR-122			Targets and downregulates TP53, thus supporting cancerous phenotype in non-small cell lung cancer cells. Downregulated in OvCa.	Validated—dual luciferase reporter assay in non-small cell lung cancer cells.	[50,51]
miR-1228			Targets and negatively regulates *TP53* in OvCa cells, thus acting as tumour suppressor. circRNA_100395 supports OvCa malignant potential through sponging miR-1228.	Validated—dual luciferase reporter assay in OvCa cells.	[52]
miR-145			Regulated by p53 and downregulated in OvCa cells and tissues, supporting cancerous phenotype.	Not applicable.	[53]
miR-200 family			p53 regulates miR-200 family (miR-200a/b/c, miR-141, miR-429). In OvCa, they show redundant tumour suppressor and oncomiR roles. Elevated serum/exosomal levels are suggested as diagnostic biomarkers.	Not applicable.	[54,55,56,57]
miR-647	MDM2 (Mouse Double Minute 2, Human Homolog)	DNA damage response; cell cycle checkpoints; apoptosis	Targets and negatively regulates MDM2 in OvCa cells. Sponged by circ-FAM53B.	Validated—dual luciferase reporter assay in OvCa cells.	[58]
miR-191	MDMX/MDM4 (Mouse Double Minute 4, Human Homolog)	DNA damage response; cell cycle checkpoints; apoptosis	MDMX 3’-UTR SNP34091 generates miR-191 site, lowering MDMX levels. This SNP occurs more often in low-grade OvCa.	Validated—dual luciferase and biotinylated miRNApull-down assays show miR-191 binds only the C, not A, variant of SNP34091 in OvCa cells.	[59]
miR-214-5p			Targets and downregulates MDMX and MDM2 in immature ovarian teratocarcinoma. LINC00324 sponges miR-214-5p to derepress MDMX and MDM2 expression.	Validated—dual luciferase reporter assay and RNA Immunoprecipitation in OvCa cells.	[60]
miR-223	ATR (Ataxia Telangiectasia and Rad3-Related Protein)	DNA damage response	Targets ATR and is sponged by circATP2B4, in pulmonary arterial smooth muscle cells. In OvCa upregulated and serves as oncomiR.	Validated—dual luciferase reporter assay and RNA immunoprecipitation in pulmonary arterial smooth muscle cells.	[61,62]
miR-1255b; miR-193b; miR-148b	BRCA1/FANCS (Breast Cancer Type 1 Susceptibility Protein); BRCA2/FANCD1 (Breast Cancer Type 2 Susceptibility Protein); RAD51/FANCR (RAD51 Recombinase)	Homologous recombination; Fanconi anaemia pathway	Deletion of miR-1255b, miR-193b, and miR-148b in OvCa correlates with genomic copy number alterations from HR proteins upregulation in G1 cell cycle phase.	Validated—dual luciferase reporter assay confirms miR-1255b binds BRCA1/2, miR-193b binds BRCA1/2 and RAD51, and miR-148b binds RAD51 in breast cancer cells.	[63]
miR-210	RAD52/RDM1 (RAD52 Homolog, DNA Repair)	Homologous recombination	Induced under hypoxia by HIF1α and targets RAD52 in breast cancer cells. Upregulated in OvCa tissues and promotes EMT and tumour growth.	Validated—dual luciferase reporter assay in breast cancer cells.	[19,64,65]
miR-146	FANCM (FA Complementation Group M)	Fanconi anaemia pathway	Targets and/or downregulates FANCM and BRCA1, induces DNA damage in cervical, gastric or breast cancer cells. Upregulated expression correlates with favourable prognosis in OvCa.	Validated—dual luciferase reporter assay confirms miR-146a binds FANCM, and miR-146a/miR-146b-5p bind BRCA1 in HeLa cells.	[66,67,68]

**Table 2:** miRNAs related to DNA repair pathways that are involved in response to OvCa treatments.

miRNA	Targeted Gene/Protein(s)	Related DNA Repair Pathway(s)	Effect	miRNA-Target Gene Interaction Validation	References
miR-211	TDP1 (Tyrosyl-DNA Phosphodiesterase-1); POLH/RAD30A (DNA Polymerase Eta); ATRX (ATP-Dependent Helicase); ERCC6L2 (ERCC Excision Repair 6 Like 2)	Single-strand break repair; translesion synthesis; transcriptional regulation and chromatin remodelling; double-strand break repair	Targets DDR genes’ transcripts, including POLH, TDP1, ATRX, and ERCC6L2 in human embryonic kidney cells. In OvCa cells, promotes platinum chemosensitivity.	Validated—dual luciferase reporter assay in human embryonic kidney cells.	[89]
let-7e	PARP1 (Poly(ADP-Ribose) Polymerase-1)	Mainly single-strand break repair, but also other repair pathways	Targets PARP1 to enhance cisplatin sensitivity in OvCa cells.	Validated—dual luciferase reporter assay in OvCa cells.	[90]
miR-216b			Downregulated in cisplatin-resistant OvCa cells; targets PARP1 to suppress malignancy, enhance cisplatin sensitivity, and inhibit tumour growth in xenograft models.	Validated—dual luciferase reporter assay in OvCa cells.	[91]
miR-100-5p	PARP1 (Poly(ADP-Ribose) Polymerase-1)ATR (Ataxia Telangiectasia And Rad3-Related Protein)CHK1/CHEK1 (Checkpoint Kinase-1)	Single-strand break repair and DNA damage response	Downregulated by PARP1 inhibitor (Olaparib) + ATR inhibitor (ceralasertib), or by CHEK1 inhibitor (MK-8776) alone treatment in OvCa cells.	Not applicable.	[92]
miR-26a-5p			Downregulated by PARP1 inhibitor (Olaparib) + ATR inhibitor (ceralasertib), or by CHEK1 inhibitor (MK-8776) alone treatment in OvCa cells.	Not applicable.	[92]
miR-33a-3p			Downregulated by PARP1 inhibitor (Olaparib) + ATR inhibitor (ceralasertib) treatment in OvCa cells.	Not applicable.	[92]
miR-99b-5p			Downregulated by PARP1 inhibitor (Olaparib) + ATR inhibitor (ceralasertib), or by CHEK1 inhibitor (MK-8776) alone treatment in OvCa cells.	Not applicable.	[92]
miR-486-5p			Downregulated by PARP1 inhibitor (Olaparib) + ATR inhibitor (ceralasertib), or by PARP1 inhibitor (Olaparib) + CHEK1 inhibitor (MK-8776), or by CHEK1 inhibitor (MK-8776) alone treatment in OvCa cells.	Not applicable.	[92]
miR-1275			Downregulated by PARP1 inhibitor (Olaparib) + CHEK1 inhibitor (MK-8776), or by CHEK1 inhibitor (MK-8776) alone treatment in OvCa cells.	Not applicable.	[92]
miR-1290			Upregulated by PARP1 inhibitor (Olaparib) + CHEK1 inhibitor (MK-8776) treatment in OvCa cells.	Not applicable.	[92]
miR-100-3p, miR-320b, miR-628-5p	CHK1/CHEK1 (Checkpoint Kinase-1)		Downregulated by CHEK1 inhibitor (MK-8776) treatment in OvCa cells.	Not applicable.	[92]
miR-379-5p	RAD18 (RAD18 E3 Ubiquitin Protein Ligase)	Translesion synthesis, homologous recombination repair	Inhibits OvCa stem cells and reduces cisplatin resistance through targeting RAD18. Also, targets PARP1 and XRCC6 in the context of premature ovarian insufficiency, where it impairs DNA repair and leads to cell death.	Validated—dual luciferase reporter assay confirmed binding with RAD18 in OvCa cells and binding with PARP1 and XRCC6 in steroidogenic human granulosa-like tumour cells.	[93,94]
miR-145			Targets RAD18 and thus controls cell sensitivity to 5-fluorouracil treatment in colorectal cancer cells. In OvCa tissues and cells, downregulated and shown to regulate targets shaping malignant potential.	Validated—dual luciferase reporter assay in human embryonic kidney cells.	[95,96,97,98]
miR-630			Downregulates RAD18 in liver cancer cells. Upregulated in OvCa tissues and cells, and supports malignant traits, as well as paclitaxel resistance.	Interaction not validated. RAD18 is downregulated by miR-630 mimic and upregulated by miR-630 inhibitor in liver cancer cells.	[99,100,101]
miR-125a-3p	BOK (BCL2 Family Apoptosis Regulator)	Apoptosis	Upregulated by combination of olaparib and cisplatin in OvCa cells. Promotes DNA damage and inhibits malignant traits of OvCa cells.	Interaction not validated. BOK is upregulated by miR-125a-3p mimic and downregulated by anti-miR-125a-3p in OvCa cells.	[102]
	PARP1 (Poly(ADP-Ribose) Polymerase-1)CHK1/CHEK1 (Checkpoint Kinase-1)	Single-strand break repair and DNA damage response	Downregulated by PARP1 inhibitor (Olaparib) + CHEK1 (MK-8776), or by CHEK1 inhibitor (MK-8776) alone treatment in OvCa cells.	Not applicable.	[92]
miR-770-5p	ERCC2/XPD (ERCC Excision Repair 2, TFIIH Core Complex Helicase Subunit/Xeroderma Pigmentosum, Complementation Group D)	Nucleotide excision repair	Downregulated in OvCa tissues of platinum-resistant patients. Predicted to target ERCC2 in OvCa cells, inducing DNA damage and increasing cisplatin sensitivity.	Interaction not validated. ERCC2 is downregulated by miR-770-5p mimic and downregulated by anti-miR-770-5p in OvCa cells.	[103]
miR-152	ERCC1 (Excision Repair Cross-Complementation Group-1)	Nucleotide excision repair	Downregulated in cisplatin-resistant OvCa tissues and inversely correlated with ERCC1 expression.	Interaction not validated. Higher miR-152 expression correlates with lower ERCC1 levels in OvCa tissues.	[104]
miR-590-5p	MSH2 (MutS Homolog-2)	Mismatch repair	Targets MSH2 in OvCa cells and thus increases cisplatin resistance.	Validated—dual luciferase reporter assay in OvCa cells.	[105]
miR-21	MSH2 (MutS Homolog-2), MSH6 (MutS Homolog-6)	Mismatch repair	Targets and downregulates MSH2 and MSH6 and thus reduces 5-FU sensitivity in colon cancer cells. Upregulated in OvCa tissues and cells, where it contributes to cisplatin resistance.	Validated—dual luciferase reporter assay in colon cancer cells.	[106,107]
miR-24	H2AX (H2A Histone Family Member X)	DNA damage response	Targets and downregulates H2AX levels in terminally differentiated blood cells, thus compromising their survival after DNA damage. miR-24-3p expression contributes to cisplatin resistance in OvCa cells. Elevated miR-24-1-5p expression supports OvCa cell proliferation and tumour growth in xenograft model.	Validated—dual luciferase reporter assay in terminally differentiated blood cells.	[108,109,110]
miR-622	Ku70/XRCC6 (X-Ray Repair Cross Complementing-6) and Ku80/XRCC5 (X-Ray Repair Cross Complementing-5)	Non-homologous end joining	Targets Ku heterodimer (Ku70/Ku80), thus inhibiting NHEJ and inducing platinum and PARP inhibitor resistance in BRCA1-mutant OvCa cells.	Validated—dual luciferase reporter and biotinylated miRNA pull-down assays in BRCA1-mutant OvCa cells.	[111]
miR-192, miR-194, miR-215	TP53/p53 (Tumour Protein P53);MDM2(Mouse Double Minute 2, Human Homolog); XPB/ERCC3 (Xeroderma Pigmentosum, Complementation Group B);XPF/ERCC4/FANCQ(Xeroderma Pigmentosum, Complementation Group F)	DNA damage response; cell cycle checkpoints; apoptosis	p53 upregulates the miR-194/215 cluster (miR-192, -194, -215), which is upregulated in mucinous OvCa, but downregulated in other subtypes. miR-194-5p targets MDM2 in OvCa, with its downregulation causing paclitaxel resistance. miR-192, -194, and -215 also target MDM2 in renal cancer cells; miR-192 targets XPB/XPF in liver cancer cells.	Validated—dual luciferase reporter assay confirms miR-192, -194, and -215 binding to MDM2 in renal cell carcinoma cells, miR-194-5p binding to MDM2 in OvCa cells, and miR-192 binding to XPB and XPF in liver cancer cells.	[112,113,114,115,116]
miR-185	ATR (Ataxia Telangiectasia And Rad3-Related Protein)	DNA damage response	Targets ATR and is downregulated upon ionising radiation to support DDR in renal cell carcinoma. Downregulated in cisplatin and paclitaxel-resistant OvCa cells.	Validated—dual luciferase reporter assay in renal cell carcinoma.	[117,118,119]
miR-9	BRCA1/FANCS (Breast Cancer Type 1 Susceptibility Protein)	DNA Damage Response; Homologous recombination; Fanconi anaemia pathway	Targets and downregulates BRCA1 and thus improves sensitivity to chemotherapeutics in OvCa cells.	Validated—dual luciferase reporter assay in OvCa cells.	[120]
miR-96	RAD51 (RAD51 Recombinase); REV1/REV1L (REV1 DNA Directed Polymerase)	Homologous recombination; translesion synthesis	Targets and downregulates REV1 and RAD51 in osteosarcoma cells, increasing sensitivity to cisplatin and PARP inhibition. Upregulated in OvCa cells and tissues and supports malignant phenotype.	Validated—dual luciferase reporter assay in osteosarcoma cells	[121,122]
miR-23a	FANCG/XRCC9 (FA Complementation Group G)	Fanconi anaemia pathway	Downregulates FANCG, thus inducing DNA damage in normal human oral fibroblasts. Overexpressed in OvCa tissue samples, and its overexpression correlates with platinum resistance.	Interaction not validated. *FANCG* is downregulated by miR-23a mimic.	[123,124,125]
miR-101	DNA-PKc/PRKDC/XRCC7 (DNA-Dependent Protein Kinase Catalytic Subunit); ATM (Ataxia Telangiectasia Mutated)	DNA damage response	Targets and downregulates DNA-PKcs and ATM, thus increasing radiosensitivity in lung and glioma cells. Downregulated in OvCa cells, and its overexpression compromises cell survival.	Validated—dual luciferase reporter assay in human embryonic kidney cells.	[126,127]
miR-506	RAD50 (Homolog of *S. cerevisiae* Rad50)RAD17 (Checkpoint Clamp Loader Component RAD17)	Homologous recombinationDNA damage response	miR-506 targets *R*AD51, and miR-506-3p targets RAD17, thus sensitising OvCa cells to chemotherapy.	Validated—dual luciferase reporter assay in HeLa or human embryonic kidney cells.	[128,129]
miR-449a	EME1 (Essential Meiotic Structure-Specific Endonuclease-1); BRCA2/FANCD1 (Breast Cancer Type 2 Susceptibility Protein/BRCA2 DNA Repair Associated); RAD51/FANCR (RAD51 Recombinase)	Homologous recombination; Fanconi anaemia pathway	Targets *E*ME1, downregulates BRCA2 and RAD51 and induces apoptosis in breast cancer cells. Potentiates PARP inhibitor effectiveness in *BRCA1*-mutated cells. Downregulated by circGFRA1-mediated sponging in OvCa.	Validated—dual luciferase reporter and RNA immunoprecipitation assays for EME1 in human embryonic kidney cells.	[130,131]

## Abbreviations

The following abbreviations are used in this manuscript:

3′UTR	3′ untranslated region
5mC	5′-methylcytosine
Ago2	Argonaute 2
ATM	Ataxia Telangiectasia Mutated
BER	base excision repair
BRCA1	BRCA1 DNA Repair Associated, also known as Breast Cancer Type 1 Susceptibility Protein
BRCA2	BRCA2 DNA Repair Associated, or Breast Cancer Type 2 Susceptibility Protein
CHEK2	Checkpoint Kinase 2
DDB2	Damage-Specific DNA Binding Protein 2
DDR	DNA damage repair
DSB	Double-strand break
FEN1	Flap Structure-Specific Endonuclease 1
HGSOC	high-grade serous ovarian carcinoma
HR	homologous recombination
IOT	immature ovarian teratocarcinoma
MBD4	Methyl-CpG Binding Domain Protein 4
miRNA	microRNA
NER	nucleotide excision repair
NHEJ	non-homologous end joining
OvCa	Ovarian cancer
PARP1	Poly(ADP-Ribose) Polymerase 1
SSB	Single-strand break

## References

[1] Webb P.M., Jordan S.J. (2024). Global epidemiology of epithelial ovarian cancer. Nat. Rev. Clin. Oncol..

[2] Caruso G., Weroha S.J., Cliby W. (2025). Ovarian Cancer: A Review. JAMA.

[3] Worzfeld T., Pogge von Strandmann E., Huber M., Adhikary T., Wagner U., Reinartz S., Muller R. (2017). The Unique Molecular and Cellular Microenvironment of Ovarian Cancer. Front. Oncol..

[4] Bowtell D.D., Bohm S., Ahmed A.A., Aspuria P.J., Bast R.C., Beral V., Berek J.S., Birrer M.J., Blagden S., Bookman M.A. (2015). Rethinking ovarian cancer II: Reducing mortality from high-grade serous ovarian cancer. Nat. Rev. Cancer.

[5] Alrosan K., Alrosan A.Z., Heilat G.B., Alrousan A.F., Gammoh O.S., Alqudah A., Madae’En S., Alrousan M.J. (2025). Treatment of ovarian cancer: From the past to the new era (Review). Oncol. Lett..

[6] Goulooze S.C., Cohen A.F., Rissmann R. (2016). Olaparib. Br. J. Clin. Pharmacol..

[7] MacGilvary N., Cantor S.B. (2024). Positioning loss of PARP1 activity as the central toxic event in BRCA-deficient cancer. DNA Repair.

[8] Seyhan A.A. (2024). Trials and Tribulations of MicroRNA Therapeutics. Int. J. Mol. Sci..

[9] Badal A.K., Nayek A., Dhar R., Karmakar S. (2025). MicroRNA nanoformulation: A promising approach to anti-tumour activity. Investig. New Drugs.

[10] Ovejero-Sanchez M., Gonzalez-Sarmiento R., Herrero A.B. (2023). DNA Damage Response Alterations in Ovarian Cancer: From Molecular Mechanisms to Therapeutic Opportunities. Cancers.

[11] Wong O.G.W., Li J., Cheung A.N.Y. (2021). Targeting DNA Damage Response Pathway in Ovarian Clear Cell Carcinoma. Front. Oncol..

[12] Ciccia A., Elledge S.J. (2010). The DNA damage response: Making it safe to play with knives. Mol. Cell.

[13] Lukacs J., Soltesz B., Penyige A., Nagy B., Poka R. (2019). Identification of miR-146a and miR-196a-2 single nucleotide polymorphisms at patients with high-grade serous ovarian cancer. J. Biotechnol..

[14] Fan Y., Fan J., Huang L., Ye M., Huang Z., Wang Y., Li Q., Huang J. (2015). Increased expression of microRNA-196a predicts poor prognosis in human ovarian carcinoma. Int. J. Clin. Exp. Pathol..

[15] Chang H., Zhang X., Li B., Meng X. (2022). PARP1 Is Targeted by miR-519a-3p and Promotes the Migration, Invasion, and Tube Formation of Ovarian Cancer Cells. Cancer Biother. Radiopharm..

[16] Wu H., Yan Y., Yuan J., Luo M., Wang Y. (2022). miR-4324 inhibits ovarian cancer progression by targeting FEN1. J. Ovarian Res..

[17] Zhao M., Ji H., Fu Q., Cheng Q., Zhang Y., Yang Y. (2021). MicroRNA-134-3p inhibits ovarian cancer progression by targeting flap structure-specific endonuclease 1 in vitro. Oncol. Rep..

[18] Srivastava A.K., Banerjee A., Cui T., Han C., Cai S., Liu L., Wu D., Cui R., Li Z., Zhang X. (2019). Inhibition of miR-328-3p Impairs Cancer Stem Cell Function and Prevents Metastasis in Ovarian Cancer. Cancer Res..

[19] Crosby M.E., Kulshreshtha R., Ivan M., Glazer P.M. (2009). MicroRNA regulation of DNA repair gene expression in hypoxic stress. Cancer Res..

[20] Meng X., Muller V., Milde-Langosch K., Trillsch F., Pantel K., Schwarzenbach H. (2016). Circulating Cell-Free miR-373, miR-200a, miR-200b and miR-200c in Patients with Epithelial Ovarian Cancer. Circulating Nucleic Acids in Serum and Plasma–CNAPS IX.

[21] Valeri N., Gasparini P., Fabbri M., Braconi C., Veronese A., Lovat F., Adair B., Vannini I., Fanini F., Bottoni A. (2010). Modulation of mismatch repair and genomic stability by miR-155. Proc. Natl. Acad. Sci. USA.

[22] Altrawy A., Talaat R.M., Nasr G.M., Badr E.A.E., Arneth R., Arneth B., Sabit H. (2025). Diagnostic and Prognostic Roles of miR-155 and miR-3173 in Breast and Ovarian Cancer: Implications for Early Detection and Personalized Treatment. Biomedicines.

[23] Liu H.Y., Zhang Y.Y., Zhu B.L., Feng F.Z., Zhang H.T., Yan H., Zhou B. (2019). MiR-203a-3p regulates the biological behaviors of ovarian cancer cells through mediating the Akt/GSK-3beta/Snail signaling pathway by targeting ATM. J. Ovarian Res..

[24] Han C., Liu Y., Wan G., Choi H.J., Zhao L., Ivan C., He X., Sood A.K., Zhang X., Lu X. (2014). The RNA-binding protein DDX1 promotes primary microRNA maturation and inhibits ovarian tumor progression. Cell Rep..

[25] Ghafour A.A., Odemis D.A., Tuncer S.B., Kurt B., Saral M.A., Erciyas S.K., Erdogan O.S., Celik B., Saip P., Yazici H. (2021). High expression level of miR-1260 family in the peripheral blood of patients with ovarian carcinoma. J. Ovarian Res..

[26] Huang Y.Z., Zhang J., Shao H.Y., Chen J.P., Zhao H.Y. (2015). MicroRNA-191 promotes osteosarcoma cells proliferation by targeting checkpoint kinase 2. Tumour Biol..

[27] Takamizawa S., Kojima J., Umezu T., Kuroda M., Hayashi S., Maruta T., Okamoto A., Nishi H. (2024). miR-146a-5p and miR-191-5p as novel diagnostic marker candidates for ovarian clear cell carcinoma. Mol. Clin. Oncol..

[28] Krishnan K., Steptoe A.L., Martin H.C., Wani S., Nones K., Waddell N., Mariasegaram M., Simpson P.T., Lakhani S.R., Gabrielli B. (2013). MicroRNA-182-5p targets a network of genes involved in DNA repair. RNA.

[29] Moskwa P., Buffa F.M., Pan Y., Panchakshari R., Gottipati P., Muschel R.J., Beech J., Kulshrestha R., Abdelmohsen K., Weinstock D.M. (2011). miR-182-mediated downregulation of BRCA1 impacts DNA repair and sensitivity to PARP inhibitors. Mol. Cell.

[30] Beg A., Parveen R., Fouad H., Yahia M.E., Hassanein A.S. (2023). Identification of Driver Genes and miRNAs in Ovarian Cancer through an Integrated In-Silico Approach. Biology.

[31] Wu J., Sun Y., Zhang P.Y., Qian M., Zhang H., Chen X., Ma D., Xu Y., Chen X., Tang K.F. (2016). The Fra-1-miR-134-SDS22 feedback loop amplifies ERK/JNK signaling and reduces chemosensitivity in ovarian cancer cells. Cell Death Dis..

[32] Wang Y., Huang J.W., Li M., Cavenee W.K., Mitchell P.S., Zhou X., Tewari M., Furnari F.B., Taniguchi T. (2011). MicroRNA-138 modulates DNA damage response by repressing histone H2AX expression. Mol. Cancer Res..

[33] Yeh Y.M., Chuang C.M., Chao K.C., Wang L.H. (2013). MicroRNA-138 suppresses ovarian cancer cell invasion and metastasis by targeting SOX4 and HIF-1alpha. Int. J. Cancer.

[34] Wang Z., Yin H., Zhang Y., Feng Y., Yan Z., Jiang X., Bukhari I., Iqbal F., Cooke H.J., Shi Q. (2014). miR-214-mediated downregulation of RNF8 induces chromosomal instability in ovarian cancer cells. Cell Cycle.

[35] Lee J.H., Park S.J., Jeong S.Y., Kim M.J., Jun S., Lee H.S., Chang I.Y., Lim S.C., Yoon S.P., Yong J. (2015). MicroRNA-22 Suppresses DNA Repair and Promotes Genomic Instability through Targeting of MDC1. Cancer Res..

[36] Wan W.N., Zhang Y.Q., Wang X.M., Liu Y.J., Zhang Y.X., Que Y.H., Zhao W.J., Li P. (2014). Down-regulated miR-22 as predictive biomarkers for prognosis of epithelial ovarian cancer. Diagn. Pathol..

[37] Pandey A.K., Zhang Y., Zhang S., Li Y., Tucker-Kellogg G., Yang H., Jha S. (2015). TIP60-miR-22 axis as a prognostic marker of breast cancer progression. Oncotarget.

[38] Lee C.H., Subramanian S., Beck A.H., Espinosa I., Senz J., Zhu S.X., Huntsman D., van de Rijn M., Gilks C.B. (2009). MicroRNA profiling of BRCA1/2 mutation-carrying and non-mutation-carrying high-grade serous carcinomas of ovary. PLoS ONE.

[39] Miles G.D., Seiler M., Rodriguez L., Rajagopal G., Bhanot G. (2012). Identifying microRNA/mRNA dysregulations in ovarian cancer. BMC Res. Notes.

[40] Welponer H., Tsibulak I., Wieser V., Degasper C., Shivalingaiah G., Wenzel S., Sprung S., Marth C., Hackl H., Fiegl H. (2020). The miR-34 family and its clinical significance in ovarian cancer. J. Cancer.

[41] Dari M.A.G., Jaberian Asl B., Dayer D., Azizidoost S., Farzaneh M., Salehi A.M. (2025). miR-34 as a Critical Regulator in Ovarian Cancer. Curr. Mol. Med..

[42] Pan W., Chai B., Li L., Lu Z., Ma Z. (2023). *p53*/MicroRNA-34 axis in cancer and beyond. Heliyon.

[43] Okada N., Lin C.P., Ribeiro M.C., Biton A., Lai G., He X., Bu P., Vogel H., Jablons D.M., Keller A.C. (2014). A positive feedback between *p53* and miR-34 miRNAs mediates tumor suppression. Genes Dev..

[44] Yamakuchi M., Ferlito M., Lowenstein C.J. (2008). miR-34a repression of SIRT1 regulates apoptosis. Proc. Natl. Acad. Sci. USA.

[45] Creighton C.J., Fountain M.D., Yu Z., Nagaraja A.K., Zhu H., Khan M., Olokpa E., Zariff A., Gunaratne P.H., Matzuk M.M. (2010). Molecular profiling uncovers a *p53*-associated role for microRNA-31 in inhibiting the proliferation of serous ovarian carcinomas and other cancers. Cancer Res..

[46] Guo J.Y., Wang X.Q., Sun L.F. (2020). MicroRNA-488 inhibits ovarian cancer cell metastasis through regulating CCNG1 and *p53* expression. Eur. Rev. Med. Pharmacol. Sci..

[47] Park S.Y., Lee J.H., Ha M., Nam J.W., Kim V.N. (2009). miR-29 miRNAs activate *p53* by targeting p85 alpha and CDC42. Nat. Struct. Mol. Biol..

[48] Teng Y., Zhang Y., Qu K., Yang X., Fu J., Chen W., Li X. (2015). MicroRNA-29B (mir-29b) regulates the Warburg effect in ovarian cancer by targeting AKT2 and AKT3. Oncotarget.

[49] Flavin R., Smyth P., Barrett C., Russell S., Wen H., Wei J., Laios A., O’Toole S., Ring M., Denning K. (2009). miR-29b expression is associated with disease-free survival in patients with ovarian serous carcinoma. Int. J. Gynecol. Cancer.

[50] Fan X., Wu X. (2019). MicroRNA-122-5p promotes the development of non-small cell lung cancer via downregulating *p53* and activating PI3K-AKT pathway. J. Balk. Union Oncol..

[51] Huang X., Luo Y., Li X. (2022). Circ_0072995 Promotes Ovarian Cancer Progression Through Regulating miR-122-5p/SLC1A5 Axis. Biochem. Genet..

[52] Li X., Lin S., Mo Z., Jiang J., Tang H., Wu C., Song J. (2020). CircRNA_100395 inhibits cell proliferation and metastasis in ovarian cancer via regulating miR-1228/*p53*/epithelial-mesenchymal transition (EMT) axis. J. Cancer.

[53] Dong R., Liu X., Zhang Q., Jiang Z., Li Y., Wei Y., Li Y., Yang Q., Liu J., Wei J.J. (2014). miR-145 inhibits tumor growth and metastasis by targeting metadherin in high-grade serous ovarian carcinoma. Oncotarget.

[54] Tamura M., Sasaki Y., Kobashi K., Takeda K., Nakagaki T., Idogawa M., Tokino T. (2015). CRKL oncogene is downregulated by *p53* through miR-200s. Cancer Sci..

[55] Chang C.J., Chao C.H., Xia W., Yang J.Y., Xiong Y., Li C.W., Yu W.H., Rehman S.K., Hsu J.L., Lee H.H. (2011). *p53* regulates epithelial-mesenchymal transition and stem cell properties through modulating miRNAs. Nat. Cell Biol..

[56] Ferneza S., Fetsych M., Shuliak R., Makukh H., Volodko N., Yarema R., Fetsych T. (2021). Clinical significance of microRNA-200 and let-7 families expression assessment in patients with ovarian cancer. Ecancermedicalscience.

[57] Cavallari I., Ciccarese F., Sharova E., Urso L., Raimondi V., Silic-Benussi M., D’Agostino D.M., Ciminale V. (2021). The miR-200 Family of microRNAs: Fine Tuners of Epithelial-Mesenchymal Transition and Circulating Cancer Biomarkers. Cancers.

[58] Sun D., Liu J., Zhou L. (2019). Upregulation of circular RNA circ-FAM53B predicts adverse prognosis and accelerates the progression of ovarian cancer via the miR-646/VAMP2 and miR-647/MDM2 signaling pathways. Oncol. Rep..

[59] Wynendaele J., Bohnke A., Leucci E., Nielsen S.J., Lambertz I., Hammer S., Sbrzesny N., Kubitza D., Wolf A., Gradhand E. (2010). An illegitimate microRNA target site within the 3’ UTR of *MDM4* affects ovarian cancer progression and chemosensitivity. Cancer Res..

[60] Chen M., Zhang M., Xie L., Wu S., Zhong Y. (2021). LINC00324 facilitates cell proliferation through competing for miR-214-5p in immature ovarian teratocarcinoma. Int. J. Mol. Med..

[61] Guo J., Zhang L., Lian L., Hao M., Chen S., Hong Y. (2020). CircATP2B4 promotes hypoxia-induced proliferation and migration of pulmonary arterial smooth muscle cells via the miR-223/ATR axis. Life Sci..

[62] Barbagallo D., Ponti D., Bassani B., Bruno A., Pulze L., Akkihal S.A., George-William J.N., Gundamaraju R., Campomenosi P. (2024). MiR-223-3p in Cancer Development and Cancer Drug Resistance: Same Coin, Different Faces. Int. J. Mol. Sci..

[63] Choi Y.E., Pan Y., Park E., Konstantinopoulos P., De S., D’Andrea A., Chowdhury D. (2014). MicroRNAs down-regulate homologous recombination in the G1 phase of cycling cells to maintain genomic stability. Elife.

[64] Li L., Huang K., You Y., Fu X., Hu L., Song L., Meng Y. (2014). Hypoxia-induced miR-210 in epithelial ovarian cancer enhances cancer cell viability via promoting proliferation and inhibiting apoptosis. Int. J. Oncol..

[65] Ding L., Zhao L., Chen W., Liu T., Li Z., Li X. (2015). miR-210, a modulator of hypoxia-induced epithelial-mesenchymal transition in ovarian cancer cell. Int. J. Clin. Exp. Med..

[66] Sundaravinayagam D., Kim H.R., Wu T., Kim H.H., Lee H.S., Jun S., Cha J.H., Kee Y., You H.J., Lee J.H. (2016). miR146a-mediated targeting of FANCM during inflammation compromises genome integrity. Oncotarget.

[67] Garcia A.I., Buisson M., Bertrand P., Rimokh R., Rouleau E., Lopez B.S., Lidereau R., Mikaelian I., Mazoyer S. (2011). Down-regulation of *BRCA1* expression by miR-146a and miR-146b-5p in triple negative sporadic breast cancers. EMBO Mol. Med..

[68] Gu Y., Zhang M., Peng F., Fang L., Zhang Y., Liang H., Zhou W., Ao L., Guo Z. (2015). The BRCA1/2-directed miRNA signature predicts a good prognosis in ovarian cancer patients with wild-type BRCA1/2. Oncotarget.

[69] Choi M., Kipps T., Kurzrock R. (2016). ATM Mutations in Cancer: Therapeutic Implications. Mol. Cancer Ther..

[70] Tian X., Xu L., Wang P. (2015). MiR-191 inhibits TNF-alpha induced apoptosis of ovarian endometriosis and endometrioid carcinoma cells by targeting DAPK1. Int. J. Clin. Exp. Pathol..

[71] Shen J., DiCioccio R., Odunsi K., Lele S.B., Zhao H. (2010). Novel genetic variants in miR-191 gene and familial ovarian cancer. BMC Cancer.

[72] Yang H., Kong W., He L., Zhao J.J., O’Donnell J.D., Wang J., Wenham R.M., Coppola D., Kruk P.A., Nicosia S.V. (2008). MicroRNA expression profiling in human ovarian cancer: miR-214 induces cell survival and cisplatin resistance by targeting PTEN. Cancer Res..

[73] Palles C., West H.D., Chew E., Galavotti S., Flensburg C., Grolleman J.E., Jansen E.A.M., Curley H., Chegwidden L., Arbe-Barnes E.H. (2022). Germline MBD4 deficiency causes a multi-tumor predisposition syndrome. Am. J. Hum. Genet..

[74] Burdova K., Hailstone R., Hanzlikova H., Caldecott K.W. (2025). FEN1 is critical for rapid single-strand break repair in G1 phase. Nucleic Acids Res..

[75] Kikuchi K., Taniguchi Y., Hatanaka A., Sonoda E., Hochegger H., Adachi N., Matsuzaki Y., Koyama H., van Gent D.C., Jasin M. (2005). Fen-1 facilitates homologous recombination by removing divergent sequences at DNA break ends. Mol. Cell. Biol..

[76] Lu X., Liu R., Wang M., Kumar A.K., Pan F., He L., Hu Z., Guo Z. (2020). MicroRNA-140 impedes DNA repair by targeting FEN1 and enhances chemotherapeutic response in breast cancer. Oncogene.

[77] Dong S., Xiao Y., Ma X., He W., Kang J., Peng Z., Wang L., Li Z. (2019). miR-193b Increases the Chemosensitivity of Osteosarcoma Cells by Promoting FEN1-Mediated Autophagy. Onco Targets Ther..

[78] Blasiak J. (2021). Single-Strand Annealing in Cancer. Int. J. Mol. Sci..

[79] Wassing I.E., Esashi F. (2021). RAD51: Beyond the break. Semin. Cell Dev. Biol..

[80] Fitch M.E., Nakajima S., Yasui A., Ford J.M. (2003). In vivo recruitment of XPC to UV-induced cyclobutane pyrimidine dimers by the *DDB2* gene product. J. Biol. Chem..

[81] Wakasugi M., Kawashima A., Morioka H., Linn S., Sancar A., Mori T., Nikaido O., Matsunaga T. (2002). DDB accumulates at DNA damage sites immediately after UV irradiation and directly stimulates nucleotide excision repair. J. Biol. Chem..

[82] Han C., Zhao R., Liu X., Srivastava A., Gong L., Mao H., Qu M., Zhao W., Yu J., Wang Q.E. (2014). *DDB2* suppresses tumorigenicity by limiting the cancer stem cell population in ovarian cancer. Mol. Cancer Res..

[83] Cui T., Srivastava A.K., Han C., Wu D., Wani N., Liu L., Gao Z., Qu M., Zou N., Zhang X. (2018). *DDB2* represses ovarian cancer cell dedifferentiation by suppressing ALDH1A1. Cell Death Dis..

[84] Collard F., Vertommen D., Fortpied J., Duester G., Van Schaftingen E. (2007). Identification of 3-deoxyglucosone dehydrogenase as aldehyde dehydrogenase 1A1 (retinaldehyde dehydrogenase 1). Biochimie.

[85] Choudhary S., Xiao T., Vergara L.A., Srivastava S., Nees D., Piatigorsky J., Ansari N.H. (2005). Role of aldehyde dehydrogenase isozymes in the defense of rat lens and human lens epithelial cells against oxidative stress. Investig. Ophthalmol. Vis. Sci..

[86] Xiao T., Shoeb M., Siddiqui M.S., Zhang M., Ramana K.V., Srivastava S.K., Vasiliou V., Ansari N.H. (2009). Molecular cloning and oxidative modification of human lens ALDH1A1: Implication in impaired detoxification of lipid aldehydes. J. Toxicol. Environ. Health Part A.

[87] Chefetz I., Grimley E., Yang K., Hong L., Vinogradova E.V., Suciu R., Kovalenko I., Karnak D., Morgan C.A., Chtcherbinine M. (2019). A Pan-ALDH1A Inhibitor Induces Necroptosis in Ovarian Cancer Stem-like Cells. Cell Rep..

[88] Grimley E., Cole A.J., Luong T.T., McGonigal S.C., Sinno S., Yang D., Bernstein K.A., Buckanovich R.J. (2021). Aldehyde dehydrogenase inhibitors promote DNA damage in ovarian cancer and synergize with ATM/ATR inhibitors. Theranostics.

[89] Wang T., Hao D., Yang S., Ma J., Yang W., Zhu Y., Weng M., An X., Wang X., Li Y. (2019). miR-211 facilitates platinum chemosensitivity by blocking the DNA damage response (DDR) in ovarian cancer. Cell Death Dis..

[90] Xiao M., Guo J., Xie L., Yang C., Gong L., Wang Z., Cai J. (2020). Let-7e Suppresses DNA Damage Repair and Sensitizes Ovarian Cancer to Cisplatin through Targeting PARP1. Mol. Cancer Res..

[91] Liu Y., Niu Z., Lin X., Tian Y. (2017). MiR-216b increases cisplatin sensitivity in ovarian cancer cells by targeting PARP1. Cancer Gene Ther..

[92] Gralewska P., Biegala L., Gajek A., Szymczak-Pajor I., Marczak A., Sliwinska A., Rogalska A. (2025). Olaparib Combined with DDR Inhibitors Effectively Prevents EMT and Affects miRNA Regulation in TP53-Mutated Epithelial Ovarian Cancer Cell Lines. Int. J. Mol. Sci..

[93] Shukla D., Mishra S., Mandal T., Charan M., Verma A.K., Khan M.M.A., Chatterjee N., Dixit A.K., Ganesan S.K., Ganju R.K. (2025). MicroRNA-379-5p attenuates cancer stem cells and reduces cisplatin resistance in ovarian cancer by regulating RAD18/Poleta axis. Cell Death Dis..

[94] Dang Y., Wang X., Hao Y., Zhang X., Zhao S., Ma J., Qin Y., Chen Z.J. (2018). MicroRNA-379-5p is associate with biochemical premature ovarian insufficiency through PARP1 and *XRCC6*. Cell Death Dis..

[95] Liu R.L., Dong Y., Deng Y.Z., Wang W.J., Li W.D. (2015). Tumor suppressor miR-145 reverses drug resistance by directly targeting DNA damage-related gene RAD18 in colorectal cancer. Tumour Biol..

[96] Hua M., Qin Y., Sheng M., Cui X., Chen W., Zhong J., Yan J., Chen Y. (2019). miR-145 suppresses ovarian cancer progression via modulation of cell growth and invasion by targeting *CCND2* and *E2F3*. Mol. Med. Rep..

[97] Zhao S., Zhang Y., Pei M., Wu L., Li J. (2021). miR-145 inhibits mitochondrial function of ovarian cancer by targeting ARL5B. J. Ovarian Res..

[98] Fredes-Garrido A., Cruz A.A., Calaf G.M., Garrido M.P., Romero C. (2025). miR-145 and miR-23b co-transfection decreases proliferation, migration, invasion and protein levels of c-MYC, ZEB1 and *ABCB1* in epithelial ovarian cancer cell lines. Mol. Med. Rep..

[99] Huan L.C., Wu J.C., Chiou B.H., Chen C.H., Ma N., Chang C.Y., Tsen Y.K., Chen S.C. (2014). MicroRNA regulation of DNA repair gene expression in 4-aminobiphenyl-treated HepG2 cells. Toxicology.

[100] Zou Y.T., Gao J.Y., Wang H.L., Wang Y., Wang H., Li P.L. (2015). Downregulation of microRNA-630 inhibits cell proliferation and invasion and enhances chemosensitivity in human ovarian carcinoma. Genet. Mol. Res..

[101] Eoh K.J., Lee S.H., Kim H.J., Lee J.Y., Kim S., Kim S.W., Kim Y.T., Nam E.J. (2018). MicroRNA-630 inhibitor sensitizes chemoresistant ovarian cancer to chemotherapy by enhancing apoptosis. Biochem. Biophys. Res. Commun..

[102] Wang Z., Pu T., Miao W., Gao Y., Gao J., Zhang X. (2025). Olaparib increases chemosensitivity by upregulating miR-125a-3p in ovarian cancer cells. Discov. Oncol..

[103] Zhao H., Yu X., Ding Y., Zhao J., Wang G., Wu X., Jiang J., Peng C., Guo G.Z., Cui S. (2016). MiR-770-5p inhibits cisplatin chemoresistance in human ovarian cancer by targeting *ERCC2*. Oncotarget.

[104] He J., Yu J.J., Xu Q., Wang L., Zheng J.Z., Liu L.Z., Jiang B.H. (2015). Downregulation of ATG14 by EGR1-MIR152 sensitizes ovarian cancer cells to cisplatin-induced apoptosis by inhibiting cyto-protective autophagy. Autophagy.

[105] Li B., Xu X., Zheng L., Jiang X., Lin J., Zhang G. (2023). MiR-590-5p promotes cisplatin resistance via targeting *hMSH2* in ovarian cancer. Mol. Biol. Rep..

[106] Valeri N., Gasparini P., Braconi C., Paone A., Lovat F., Fabbri M., Sumani K.M., Alder H., Amadori D., Patel T. (2010). MicroRNA-21 induces resistance to 5-fluorouracil by down-regulating human DNA MutS homolog 2 (*hMSH2*). Proc. Natl. Acad. Sci. USA.

[107] Jiang N.J., Yin Y.N., Lin J., Li W.Y., Long D.R., Mei L. (2023). MicroRNA-21 in gynecological cancers: From molecular pathogenesis to clinical significance. Pathol. Res. Pract..

[108] Lal A., Pan Y., Navarro F., Dykxhoorn D.M., Moreau L., Meire E., Bentwich Z., Lieberman J., Chowdhury D. (2009). miR-24-mediated downregulation of H2AX suppresses DNA repair in terminally differentiated blood cells. Nat. Struct. Mol. Biol..

[109] Liu W., Wang S., Zhou S., Yang F., Jiang W., Zhang Q., Wang L. (2017). A systems biology approach to identify microRNAs contributing to cisplatin resistance in human ovarian cancer cells. Mol. Biosyst..

[110] Zhang W., Fei J., Yu S., Shen J., Zhu X., Sadhukhan A., Lu W., Zhou J. (2018). LINC01088 inhibits tumorigenesis of ovarian epithelial cells by targeting miR-24-1-5p. Sci. Rep..

[111] Choi Y.E., Meghani K., Brault M.E., Leclerc L., He Y.J., Day T.A., Elias K.M., Drapkin R., Weinstock D.M., Dao F. (2016). Platinum and PARP Inhibitor Resistance Due to Overexpression of MicroRNA-622 in BRCA1-Mutant Ovarian Cancer. Cell Rep..

[112] Jones M., Lal A. (2012). MicroRNAs, wild-type and mutant *p53*: More questions than answers. RNA Biol..

[113] Agostini A., Brunetti M., Davidson B., Trope C.G., Eriksson A.G.Z., Heim S., Panagopoulos I., Micci F. (2018). The microRNA miR-192/215 family is upregulated in mucinous ovarian carcinomas. Sci. Rep..

[114] Khella H.W., Bakhet M., Allo G., Jewett M.A., Girgis A.H., Latif A., Girgis H., Von Both I., Bjarnason G.A., Yousef G.M. (2013). miR-192, miR-194 and miR-215: A convergent microRNA network suppressing tumor progression in renal cell carcinoma. Carcinogenesis.

[115] Nakamura K., Sawada K., Miyamoto M., Kinose Y., Yoshimura A., Ishida K., Kobayashi M., Shimizu A., Nakatsuka E., Hashimoto K. (2019). Downregulation of miR-194-5p induces paclitaxel resistance in ovarian cancer cells by altering MDM2 expression. Oncotarget.

[116] Xie Q.H., He X.X., Chang Y., Sun S.Z., Jiang X., Li P.Y., Lin J.S. (2011). MiR-192 inhibits nucleotide excision repair by targeting *ERCC3* and *ERCC4* in HepG2.2.15 cells. Biochem. Biophys. Res. Commun..

[117] Wang J., He J., Su F., Ding N., Hu W., Yao B., Wang W., Zhou G. (2013). Repression of ATR pathway by miR-185 enhances radiation-induced apoptosis and proliferation inhibition. Cell Death Dis..

[118] Xiang Y., Ma N., Wang D., Zhang Y., Zhou J., Wu G., Zhao R., Huang H., Wang X., Qiao Y. (2014). MiR-152 and miR-185 co-contribute to ovarian cancer cells cisplatin sensitivity by targeting DNMT1 directly: A novel epigenetic therapy independent of decitabine. Oncogene.

[119] Liu Y., Shen Z., Wei X., Gu L., Zheng M., Zhang Y., Cheng X., Fu Y., Lu W. (2023). CircSLC39A8 attenuates paclitaxel resistance in ovarian cancer by regulating the miR-185-5p/BMF axis. Transl. Oncol..

[120] Sun C., Li N., Yang Z., Zhou B., He Y., Weng D., Fang Y., Wu P., Chen P., Yang X. (2013). miR-9 regulation of *BRCA1* and ovarian cancer sensitivity to cisplatin and PARP inhibition. J. Natl. Cancer Inst..

[121] Wang Y., Huang J.W., Calses P., Kemp C.J., Taniguchi T. (2012). MiR-96 downregulates REV1 and RAD51 to promote cellular sensitivity to cisplatin and PARP inhibition. Cancer Res..

[122] Liu B., Zhang J., Yang D. (2019). miR-96-5p promotes the proliferation and migration of ovarian cancer cells by suppressing Caveolae1. J. Ovarian Res..

[123] Tsai Y.S., Lin C.S., Chiang S.L., Lee C.H., Lee K.W., Ko Y.C. (2011). Areca nut induces miR-23a and inhibits repair of DNA double-strand breaks by targeting *FANCG*. Toxicol. Sci..

[124] Todeschini P., Salviato E., Romani C., Raimondi V., Ciccarese F., Ferrari F., Tognon G., Marchini S., D’Incalci M., Zanotti L. (2021). Comprehensive Profiling of Hypoxia-Related miRNAs Identifies miR-23a-3p Overexpression as a Marker of Platinum Resistance and Poor Prognosis in High-Grade Serous Ovarian Cancer. Cancers.

[125] Su L., Liu M. (2018). Correlation analysis on the expression levels of microRNA-23a and microRNA-23b and the incidence and prognosis of ovarian cancer. Oncol. Lett..

[126] Yan D., Ng W.L., Zhang X., Wang P., Zhang Z., Mo Y.Y., Mao H., Hao C., Olson J.J., Curran W.J. (2010). Targeting DNA-PKcs and ATM with miR-101 sensitizes tumors to radiation. PLoS ONE.

[127] Wei M., Jin H., Yang S., Li Z., Wang X., Li L., Jia Y., Cui M. (2021). MicroRNA-101 inhibits growth and metastasis of human ovarian cancer cells by targeting PI3K/AKT. Arch. Med. Sci..

[128] Liu G., Yang D., Rupaimoole R., Pecot C.V., Sun Y., Mangala L.S., Li X., Ji P., Cogdell D., Hu L. (2015). Augmentation of response to chemotherapy by microRNA-506 through regulation of RAD51 in serous ovarian cancers. J. Natl. Cancer Inst..

[129] Bagnoli M., Nicoletti R., Valitutti M., Rizzo A., Napoli A., Montalvao De Azevedo R., Tomassetti A., Mezzanzanica D. (2022). Impairment of RAD17 Functions by miR-506-3p as a Novel Synthetic Lethal Approach Targeting DNA Repair Pathways in Ovarian Cancer. Front. Oncol..

[130] Vajen B., Bhowmick R., Greiwe L., Schaffer V., Eilers M., Reinkens T., Stalke A., Schmidt G., Fiedler J., Thum T. (2022). MicroRNA-449a Inhibits Triple Negative Breast Cancer by Disturbing DNA Repair and Chromatid Separation. Int. J. Mol. Sci..

[131] Liu J., Yu F., Wang S., Zhao X., Jiang F., Xie J., Deng M. (2019). circGFRA1 Promotes Ovarian Cancer Progression By Sponging miR-449a. J. Cancer.

[132] Chin Sang C., Moore G., Tereshchenko M., Zhang H., Nosella M.L., Dasovich M., Alderson T.R., Leung A.K.L., Finkelstein I.J., Forman-Kay J.D. (2024). PARP1 condensates differentially partition DNA repair proteins and enhance DNA ligation. EMBO Rep..

[133] Pazzaglia S., Pioli C. (2019). Multifaceted Role of PARP-1 in DNA Repair and Inflammation: Pathological and Therapeutic Implications in Cancer and Non-Cancer Diseases. Cells.

[134] Zhu T., Zheng J.Y., Huang L.L., Wang Y.H., Yao D.F., Dai H.B. (2023). Human PARP1 substrates and regulators of its catalytic activity: An updated overview. Front. Pharmacol..

[135] Liu J., Matulonis U.A. (2025). Update on PARP inhibitors for the treatment of ovarian cancer. Clin. Adv. Hematol. Oncol..

[136] Prakash R., Zhang Y., Feng W., Jasin M. (2015). Homologous recombination and human health: The roles of *BRCA1*, *BRCA2*, and associated proteins. Cold Spring Harb. Perspect. Biol..

[137] Nielsen F.C., van Overeem Hansen T., Sorensen C.S. (2016). Hereditary breast and ovarian cancer: New genes in confined pathways. Nat. Rev. Cancer.

[138] Chen C.C., Feng W., Lim P.X., Kass E.M., Jasin M. (2018). Homology-Directed Repair and the Role of *BRCA1*, *BRCA2*, and Related Proteins in Genome Integrity and Cancer. Annu. Rev. Cancer Biol..

[139] Arun B., Couch F.J., Abraham J., Tung N., Fasching P.A. (2024). BRCA-mutated breast cancer: The unmet need, challenges and therapeutic benefits of genetic testing. Br. J. Cancer.

[140] Ma X., Fu H., Sun C., Wu W., Hou W., Zhou Z., Zheng H., Gong Y., Wu H., Qin J. (2024). RAD18 O-GlcNAcylation promotes translesion DNA synthesis and homologous recombination repair. Cell Death Dis..

[141] Hoege C., Pfander B., Moldovan G.L., Pyrowolakis G., Jentsch S. (2002). RAD6-dependent DNA repair is linked to modification of PCNA by ubiquitin and SUMO. Nature.

[142] Zeng X., Zheng W., Sheng Y., Ma H. (2022). *UBE2B* promotes ovarian cancer growth via promoting RAD18 mediated *ZMYM2* monoubiquitination and stabilization. Bioengineered.

[143] Wassing I.E., Graham E., Saayman X., Rampazzo L., Ralf C., Bassett A., Esashi F. (2021). The RAD51 recombinase protects mitotic chromatin in human cells. Nat. Commun..

[144] Wang Q., Goldstein M., Alexander P., Wakeman T.P., Sun T., Feng J., Lou Z., Kastan M.B., Wang X.F. (2014). Rad17 recruits the MRE11-RAD50-NBS1 complex to regulate the cellular response to DNA double-strand breaks. EMBO J..

[145] Kim H., Xu H., George E., Hallberg D., Kumar S., Jagannathan V., Medvedev S., Kinose Y., Devins K., Verma P. (2020). Combining PARP with ATR inhibition overcomes PARP inhibitor and platinum resistance in ovarian cancer models. Nat. Commun..

[146] Kim H., George E., Ragland R., Rafail S., Zhang R., Krepler C., Morgan M., Herlyn M., Brown E., Simpkins F. (2017). Targeting the ATR/CHK1 Axis with PARP Inhibition Results in Tumor Regression in BRCA-Mutant Ovarian Cancer Models. Clin. Cancer Res..

[147] Ma Z., Dang R., Wu G. (2025). KU60019 inhibits ovarian cancer progression by targeting DGAT1/has-miR-1273g-3p axis. PLoS ONE.

[148] Gupta S., Gellert M., Yang W. (2011). Mechanism of mismatch recognition revealed by human MutSbeta bound to unpaired DNA loops. Nat. Struct. Mol. Biol..

[149] Ren J., Wu Y., Wang Y., Zhao Y., Li Y., Hao S., Lin L., Zhang S., Xu X., Wang H. (2021). CtIP suppresses primary microRNA maturation and promotes metastasis of colon cancer cells in a xenograft mouse model. J. Biol. Chem..

[150] Kawai S., Amano A. (2012). *BRCA1* regulates microRNA biogenesis via the DROSHA microprocessor complex. J. Cell Biol..

[151] (2024). What will it take to get miRNA therapies to market?. Nat. Biotechnol..

[152] Zhang S., Lu Z., Unruh A.K., Ivan C., Baggerly K.A., Calin G.A., Li Z., Bast R.C., Le X.F. (2015). Clinically relevant microRNAs in ovarian cancer. Mol. Cancer Res..

[153] Chorley B.N., Atabakhsh E., Doran G., Gautier J.C., Ellinger-Ziegelbauer H., Jackson D., Sharapova T., Yuen P.S.T., Church R.J., Couttet P. (2021). Methodological considerations for measuring biofluid-based microRNA biomarkers. Crit. Rev. Toxicol..

[154] Condrat C.E., Thompson D.C., Barbu M.G., Bugnar O.L., Boboc A., Cretoiu D., Suciu N., Cretoiu S.M., Voinea S.C. (2020). miRNAs as Biomarkers in Disease: Latest Findings Regarding Their Role in Diagnosis and Prognosis. Cells.

